# Initial Distance‐Dependent Mean Force Drives Synaptic Vesicle Motion Toward Fusion Sites in Stimulated Hippocampal Neurons

**DOI:** 10.1002/advs.202513823

**Published:** 2025-12-19

**Authors:** Gyunam Park, Ji‐Hyun Kim, Hunki Lee, Chungwon Park, Sidong Chen, Luke Bates, Jaeyoung Sung, Hyokeun Park

**Affiliations:** ^1^ Global Science Research Center for Systems Chemistry Chung‐Ang University Seoul 06974 South Korea; ^2^ Creative Research Initiative Center for Chemical Dynamics in Living Cells Chung‐Ang University Seoul 06974 South Korea; ^3^ Department of Chemistry Chung‐Ang University Seoul 06974 South Korea; ^4^ Max‐Planck Institute for Molecular Biomedicine Röntgenstraße 20 48149 Münster Germany; ^5^ Division of Life Science The Hong Kong University of Science and Technology Clear Water Bay Kowloon Hong Kong SAR China; ^6^ Department of Computer Science UKP Lab and Hessian Center for AI Technical University of Darmstadt Hochschulstraße 10 64289 Darmstadt Germany; ^7^ Department of Physics The Hong Kong University of Science and Technology Clear Water Bay Kowloon Hong Kong SAR China; ^8^ State Key Laboratory of Nervous System Disorders The Hong Kong University of Science and Technology Clear Water Bay Kowloon Hong Kong SAR China

**Keywords:** diffusion, exocytosis, hippocampus, synaptic vesicles, transport

## Abstract

Neuronal communication occurs through transport and exocytosis of synaptic vesicles (SVs). However, their dynamics during neuronal stimulation remains poorly understood. Here, real‐time, 3D motion of individual SVs undergoing exocytosis in presynaptic terminals is quantitatively investigated. SVs are categorized into two types: Type I showing confined motion near fusion sites until exocytosis and Type II SVs exhibiting unconfined motion before tethering and exocytosis. Type II SVs have a broader fusion time distribution with a higher mean value than Type I SVs. Electrical stimulation increases the straightness of the Type II trajectories toward their fusion sites approximately tenfold. To quantify the straightness of the SV trajectories, a straightness parameter is introduced and its relationship to the mean force exerted on SVs is established. Interestingly, the straightness parameter, and hence mean velocity, increase in a sigmoidal manner with the initial distances of Type II SVs from their fusion sites upon stimulation, which results in a counterintuitive non‐monotonic dependence of their fusion time on the initial distances. A quantitative model is presented that simultaneously explains various experimental results regarding SV transport and fusion dynamics. This work offers new insights into mysterious SV motion at presynaptic terminals and its consequences on synaptic transmission of stimulated neurons.

## Graphical abstract

Synaptic vesicle (SV) motion and exocytosis dynamics in the presynaptic terminals of live neurons are intimately linked to synaptic transmission during neuronal activity. In this work, it is revealed that, during electrical stimulation, SVs located farther from their fusion sites exhibit greater straightness and experience larger mean forces directed toward the fusion sites compared to those positioned closer.

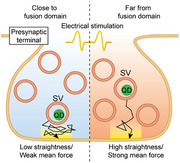

## 1 Introduction

The brain functions through neuronal communication.^[^ ^1^ ^]^ The neuronal communication is mediated by intracellular motion of synaptic vesicles (SVs) and the subsequent release of neurotransmitters contained in these vesicles at presynaptic terminals through a tightly regulated process known as exocytosis.^[^ ^2^, ^3^ ^]^ Extensive research has highlighted the importance of SV motion for exocytosis, which is involved in synaptic transmission,^[^ ^4^, ^5^, ^6^, ^7^, ^8^, ^9^, ^10^ ^]^ synaptic plasticity,^[^ ^11^ ^]^ and neurodegenerative diseases.^[^ ^12^ ^]^ In particular, the transport of SVs is crucial for supplying them to the active zone or the fusion domain in the presynaptic terminal, thereby sustaining synaptic transmission during prolonged neuronal activity.^[^ ^13^, ^14^ ^]^ The early measurements using the fluorescence recovery after photobleaching (FRAP) of fluorescently labeled SVs indicated that SVs remained largely restricted and exhibited limited mobility within the presynaptic terminal.^[^ ^15^, ^16^ ^]^ However, it was later observed that SVs in the recycling pool are mobile in the presynaptic terminal, while SVs in the reserve pool are immobile.^[^ ^17^ ^]^ Recent electron microscopy images revealed that many endocytosed SVs are located hundreds of nanometers away from the active zone,^[^ ^18^, ^19^ ^]^ raising the possibility that these SVs may perform biased motion toward their fusion domain for effective synaptic transmission.

Despite the important roles SV transport plays in physiology,^[^ ^7^, ^20^ ^]^ dynamics of individual SV transport during neuronal activities have remained elusive, primarily due to technical challenges in tracking of the small‐sized SVs, which have a diameter of ≈ 40 nm, well below the resolution of light microscopy.^[^ ^7^, ^9^, ^21^ ^]^ The recent development of real‐time, 3D tracking of single SVs in living hippocampal neurons, with tens of nanometer accuracy, has provided new insight into the role of the SV dynamics on synaptic transmission.^[^ ^6^, ^8^, ^9^, ^22^ ^]^ However, the transport and fusion dynamics of individual SVs have yet to be quantitatively understood.^[^ ^10^ ^]^

In order to address these challenges, we quantitatively investigated the real‐time, 3D motion of individual SVs and ensuing exocytosis dynamics in the central synapses in living hippocampal neurons, which play a central role in learning and memory.^[^ ^23^, ^24^ ^]^ We observed that SV motion in the neurons dramatically changes upon electrical stimulation. Specifically, during electrical stimulation, the trajectory straightness and mean velocity of SVs toward their fusion sites increase by approximately tenfold compared to their values before stimulation. Interestingly, SVs located farther from the fusion domain tend to drift faster toward the fusion domain upon electrical stimulation, exhibiting higher straightness in their trajectories. This initial distance‐dependent straightness, or mean velocity, of SV trajectories results in a counterintuitive weak correlation between the fusion times and initial distances from the fusion domain. We introduced a straightness parameter that measures the straightness of SV trajectories toward their fusion sites and established its relationship with the mean force exerted on the SVs and thermal energy. We also proposed a novel model of SV transport in neurons that simultaneously explains various experimental results of electric stimulation‐dependent SV motion.

## 2 Results

### 2.1 Two Types of Synaptic Vesicle Motion

**Figure 1:**
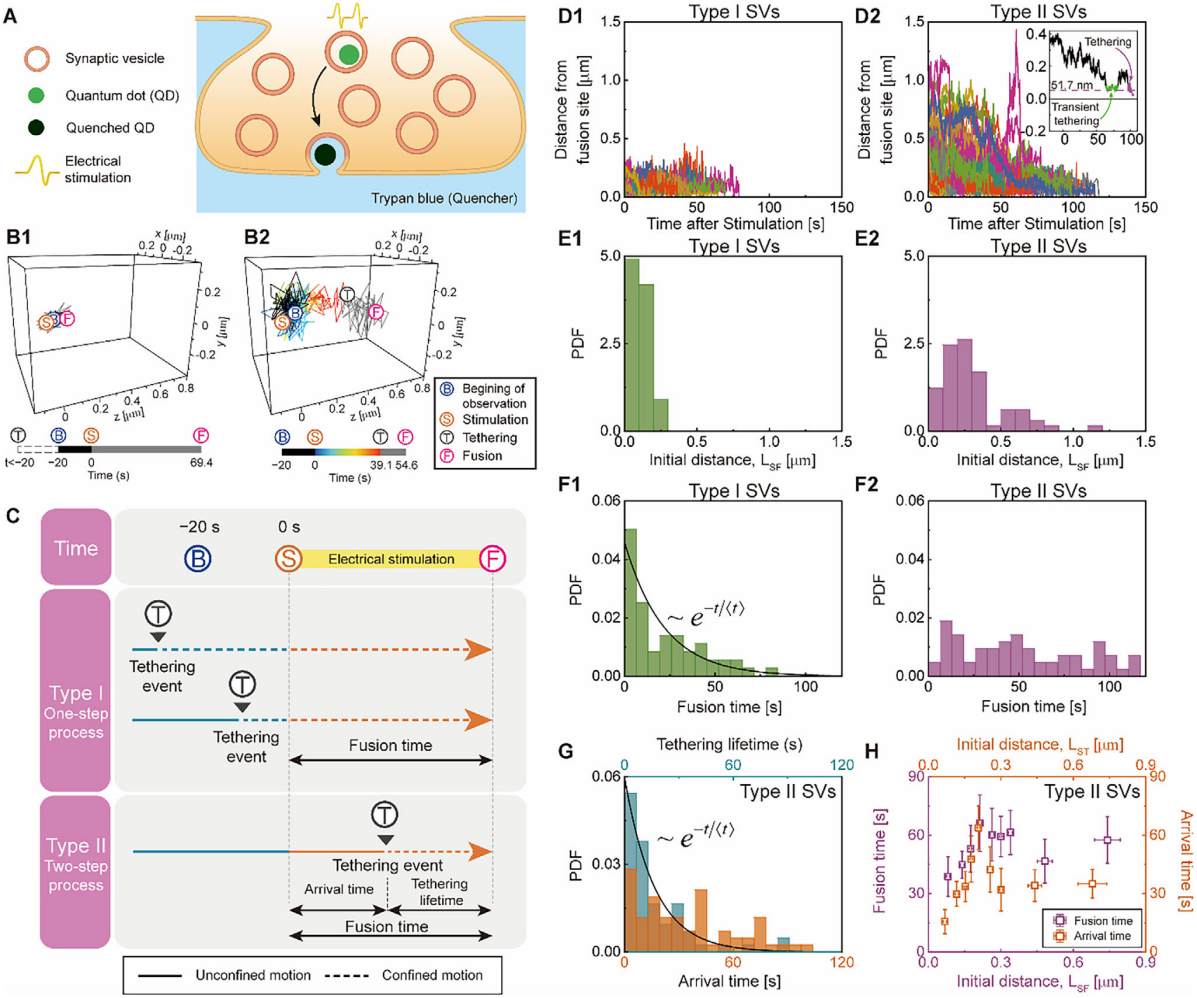
Classification of SVs and their type‐specific motion. A) Schematic representation of SVs undergoing exocytosis in the presynaptic terminal during electrical stimulation. SVs containing a bright quantum dot (QD) move toward fusion sites in the plasma membrane during electrical stimulation and undergo fusion with the membrane. During fusion, the QD is quenched by external solution containing trypan blue (quencher) and loses its fluorescence. B) Representative trajectories of B1) Type I SV, an SV already tethered to the fusion domain before electrical stimulation, showing confined motion until the fusion event and B2) Type II SV, an untethered SV undergoing unconfined motion toward its fusion site at the onset of stimulation and subsequent tethering to the fusion domain. After tethering, Type II SVs exhibit confined motion similar to that of Type I SVs. C) Timelines and fusion processes of Type I and Type II SVs. D) Time‐dependent distances of SVs from their fusion sites after electrical stimulation: D1) Type I SVs (*n* = 55) and D2) Type II SVs (*n* = 65). The inset in D2) shows an example trajectory showing a detethering event, flanked by the tethering event (green line) and the final tethering before fusion (purple line). The pink dashed line represents the mean distance during the tethering. E) Distribution of the initial distances of SVs from their fusion sites, or the distances *L*_SF_ between the site at the onset of electrical stimulation (Ⓢ) and the fusion site (Ⓕ). F) Distribution of fusion times. G) Distribution of arrival times and tethering lifetimes for Type II SVs. In F,G), the solid line represents an exponential distribution with the mean lifetime, 〈*τ*〉, in each type: 〈*τ*〉_Type I_ = 21.7 s and 〈*τ*〉_Type II_ = 16.7 s. The fusion times of Type I SVs have a distribution similar to the tethering lifetimes of Type II SVs (*p* = 0.24, Kolmogorov‐Smirnov (KS)‐test), while showing a significant difference from the distribution of fusion times of Type II SVs (*p* < 0.001, KS‐test). H) Initial distance *L*_SF_ and *L*_ST_ dependence of the fusion time and arrival time for Type II SVs. *L*_ST_ denotes the distance from the site at the onset of electrical stimulation (Ⓢ) to the tethering site (Ⓣ). The error bars represent the standard error of the mean calculated from individual bins, each of which includes nine data points, with partial overlap between neighboring bins due to the moving average.

To investigate the SV transport and fusion dynamics in the presynaptic terminal of neurons under stimulation, we tracked 3D trajectories of 120 individual SVs (*n* = 52 experiments) undergoing fusion under electrical field stimulation in presynaptic terminals of the central synapses of living primary hippocampal neurons (see details in Experimental Section) (**Figure** **1**A). For this purpose, we loaded intracellular SVs with bright quantum dots (QDs) conjugated to antibodies targeting the luminal domain of synaptotagmin 1 (Syt1), a transmembrane protein embedded in the SV membrane. To do that, we stimulated neurons with 1200 electrical stimuli at frequency of 10 Hz in the presence of these conjugated QDs. After QDs were endocytosed into neurons and encapsulated in SVs, the 3D trajectories of SVs containing QDs were measured for 20 s without stimulation and then for the next 120 s with electrical stimulation at a frequency of 10 Hz. The spatial and temporal resolutions of our 3D tracking are tens of nanometers and 0.1 s, respectively (see also details in Experimental Section). Previous studies have demonstrated that loading SVs with QDs does not affect their exocytosis or motion in living neurons.^[^ ^6^, ^22^ ^]^

We categorized SVs into two distinct types based on their motion during electrical stimulation. Some SVs undergo only confined motion near fusion sites during stimulation before exocytosis, designated by Type I (Figure 1B1). In contrast, the others, designated by Type II, exhibit unconfined motion within the presynaptic terminal during stimulation and the subsequent confined motion around the fusion sites before exocytosis (Figure 1B2,C). Among the total 120 SVs, 55 belonged to Type I, and 65 belonged to Type II. For Type II SVs, the onset of confined motion is designated by tethering. The 3D domain where SV trajectories span between the tethering and fusion events is referred to as the fusion domain (see Text S1 and Figure S1 and S2, Supporting Information). The size of the fusion domain could be estimated by the radius of gyration of an SV trajectory between the tethering and fusion times (see Figure S2B, Supporting Information), which is found to be 25.9 ± 1.23 nm (*n* = 120) (average ± standard error of the mean (SEM)). Exocytosis of Type I SVs can be approximated by a single‐step Poisson process, whereas exocytosis of Type II SVs is a multi‐step process comprising the transport toward the fusion domain, the subsequent confined motion in the fusion domain, and the final membrane fusion (Figure 1C,F).

The trajectory lengths of SVs and their distances from fusion sites at time zero, or at the onset of electrical stimulation, are strongly dependent on the SV type (Figure 1D). Type II SVs have longer trajectories and larger initial distances than Type I SVs, which results because Type I SVs are located closer to their fusion sites than Type II SVs at time zero. The value of the mean initial distance is estimated to be 104 ± 7.85 nm (*n* = 55) for Type I and 309 ± 26.3 nm (*n* = 65) for Type II. Type II SVs have a wider distribution of the initial distance from the fusion sites than Type I SVs (Figure 1E). Approxmiately 10% of Type II SV trajectories exhibited detethering after the first tethering and subsequent retethering events before the final fusion (see inset of Figure 1D2), where detethering designates leaving the fusion domain without exocytosis. The inset of Figure 1D2 shows an example trajectory showing a transient tethering and detethering event. This detethering event is reminiscent of the previously reported depriming of SVs.^[^ ^25^, ^26^ ^]^

Type II SVs have fusion times far greater than Type I SVs. The fusion time (FT) of an SV is defined as the time between the onset of neuronal stimulation and fusion of the SV with the plasma mem brane. The mean FT was 21.7 ± 2.79 s (Figure 1F1) for Type I SVs and 51.7 ± 4.15 s (Figure 1F2) for Type II SVs. Type II SVs have a longer FT due to their larger initial distance from the fusion sites compared to Type I SVs. Consequently, the FT distributions of the two types of SVs are significantly different from each other (Kolmogorov‐Smirnov (KS) test, *p* < 0.001). The FT of Type I SVs exhibits a quasi‐exponential distribution, with the relative variance given by 0.90. This implies that exocytosis of Type I SV is similar to a single‐step Poisson process, or simple rate process, for which the relative variance of the fusion time is unity. In contrast, the FT of Type II SVs exhibits a nearly uniform distribution with sub‐Poisson characteristics across our observation time range. This indicates that exocytosis of Type II SVs is not a single‐step Poisson process but a multi‐step process. The FT of Type II SVs can be decomposed into the time taken for SVs to arrive at and be tethered to the fusion domain, which is designated as the arrival time, and the time taken for the tethered SVs to undergo exocytosis, which is designated as tethering lifetime (see Figure 1C). The mean arrival time of Type II SVs is 35.0 ± 3.56 s and their arrival times follow a sub‐Poisson distribution with the relative variance of 0.6 (Figure 1G). On the other hand, the mean tethering lifetime of Type II SVs is about half the mean arrival time, given by 16.7 ± 2.37 s, and the tethering lifetime has a quasi‐exponential distribution with the relative variance given by 1.3. Therefore, it is the arrival time rather than tethering lifetime that causes the FT of Type II SVs to have a sub‐Poisson distribution.

The tethering lifetime of Type II SVs is similar to the fusion time of Type I SVs. We found that the difference between the tethering lifetime distribution of Type II SVs and the FT distribution of Type I SVs is not statistically significant (KS test, *p* = 0.24). Moreover, the tethering lifetime of Type II SVs has a quasi‐exponential distribution (Figure 1G) similar to the FT distribution of Type I SVs (Figure 1F1). These results suggest that, after being tethered, Type II SVs have similar exocytosis dynamics to Type I SVs.

### 2.2 Counterintuitive Weak Correlation between Fusion Time and Initial Distance of Type II SVs

**Figure 2:**
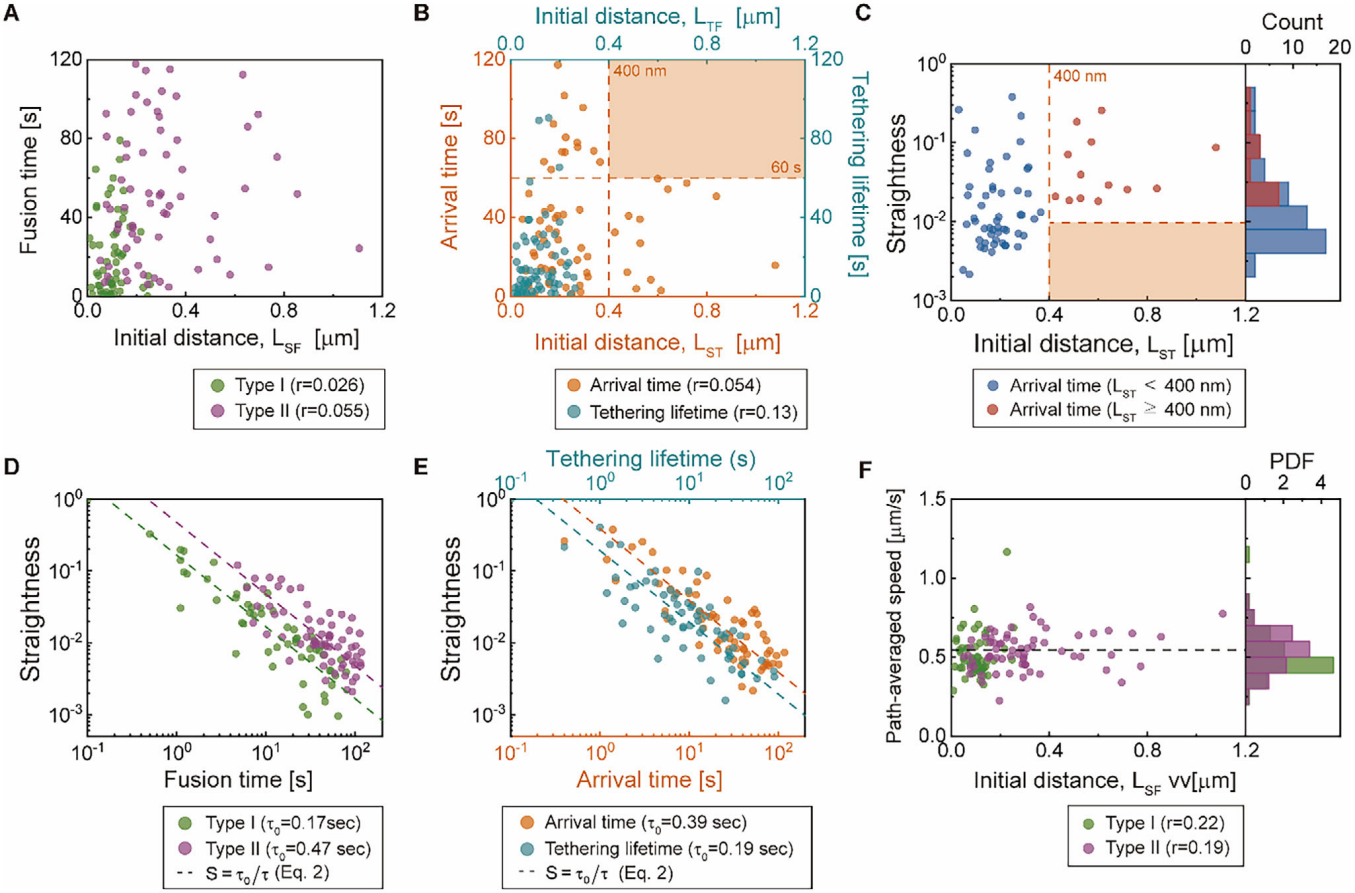
Weak correlation between initial distance and fusion time of SVs resulting from initial distance‐dependent straightness parameter. A) Correlation between fusion times and initial distances *L*_SF_ for Type I (green, *n* = 55) and Type II SVs (purple, *n* = 65). B) Correlation between the initial distance *L*_ST_ from the site at the onset of electrical stimulation (Ⓢ) to the tethering site (Ⓣ) and the arrival time (orange), and between the distance LTF from the tethering site (Ⓣ) to the fusion site (Ⓕ) and the tethering lifetime (cyan), for Type II SVs. C) Straightness of the trajectories of Type II SVs arriving at their tethering sites: SVs with *L*_ST_ less than 400 nm (blue) and SVs with *L*_ST_ greater than 400 nm (red). The histograms of straightness parameter show that SV trajectories with initial distances greater than 400 nm have straightness parameter values no less than 0.02 (*p* = 2.0 × 10^−4^, KS‐test). D) Relationship between the straightness and the fusion time for Type I SVs (green) or for Type II SVs (purple). E) Relationship between the straightness and the arrival time (orange), or the tethering lifetime (cyan), for Type II SVs. F) Path‐averaged speed *v_p_* estimated from the fusion trajectories of Type I SVs (green) and Type II SVs (purple). The path‐averaged speed histograms for Type I and Type II SVs show a narrow distribution centered ≈ 540 nm s^−1^ (dashed horizontal line). In D, E), the dashed line represents the best fit of Equation (2) (see Table 1).

Our analyses reveal that fusion time does not simply increase with the initial distance for either type of SV. The FT of Type II SVs exhibits a non‐monotonic dependence on the initial distance (Figure 1H), whereas the FT of Type I SVs is largely independent of their initial distance (**Figure** **2**A). The Pearson correlation coefficients (*r*) between the FT and initial distance are only ≈ 0.026 for Type I SVs and 0.055 for Type II SVs (Figure 2A). For Type I SVs, the weak correlation with initial distance as well as with the quasi‐exponential distribution of the FT suggests that their fusion process is more akin to a one‐step activation‐controlled process. Similarly, the tethering lifetime of Type II SVs shows a weak correlation with the initial distance (*r* = 0.13) and exhibits a quasi‐exponential distribution. However, the overall fusion process of Type II SVs is dominantly contributed to by their transport toward the fusion domain. The weak correlation between the initial distance and the overall fusion time of Type II SVs cannot be explained by the conventional diffusion‐controlled reaction model^[^ ^27^ ^]^ or other previously reported models,^[^ ^28^, ^29^ ^]^ where a particle located farther from a target is likely to take longer time to reach the target. However, as shown in Figure 2A, Type II SVs initially located closer to their fusion sites often have similar fusion times as those initially located farther away. This counterintuitive weak correlation between the FT and initial distance of Type II SVs arises because their arrival times do not simply increase with the initial distance. We confirmed this is the case; the Pearson correlation value between the arrival times and initial distance of Type II SVs is only ≈ 0.054 (see Figure 2B). Notably, we observed that SVs with initial distances less than 400 nm from their tethering sites (a dashed vertical line in Figure 2B) have a wide range of arrival times, reaching up to 120 s. In contrast, SVs with initial distances greater than 400 nm have arrival times less than 60 s. This observation implies that the transport mechanisms of Type II SVs are heterogeneous and dependent on the initial distances from their fusion site. However, we found that the average speed of SVs along their trajectories does not show any systematic dependence on their initial distances, as shown later in this work.

To understand the counterintuitive weak correlation between the initial distance and arrival time of Type II SVs, we investigated how the straightness of SV trajectories depends on the initial distance from the tethering site. For this purpose, we introduced the straightness parameter (*S*) defined as the ratio of the initial distance to the pathlength, which was first used in zoology to characterize animal migration trajectories:^[^ ^30^, ^31^ ^]^

(1) $$S=\frac{L}{\Lambda }$$

where *L* and Λ denote the end‐to‐end distance and the pathlength of SV trajectory, respectively. For a trajectory consisting of *N* discrete data points, *L* is the distance between the first point and the last point, while Λ is the total sum of *N*−1 distances between adjacent data points in the trajectory. The straightness parameter quantifies how directly SVs move toward the target point. The straightness has the value of unity (its maximum value) when an SV performs purely unidirectional, ballistic motion toward a fusion site, but is a stochastic variable with probable values less than unity when an SV undergoes random motion. The value of straightness parameter increases with the mean force exerted on SVs toward their fusion sites, as shown later in this work.

Straightness parameter for the trajectories of Type II SVs during their motion to the fusion domain is found to have a significant positive correlation with the initial distance. The corresponding Pearson correlation value is found to be ≈ 0.36 (Figure 2C), which implies that SVs located farther from the fusion domain move more straightforward toward the fusion domain. Interestingly, SV trajectories with initial distances less than 400 nm include those with straightness parameter values smaller than 10^−2^ whereas SV trajectories with initial distances greater than 400 nm do not (Figure 2C, KS test, *p* < 0.001).

The fusion time is strongly dependent on the straightness parameter for both SV types. The straightness parameter has a strong negative correlation with the fusion time for both SV types (Figure 2D and *r* = −0.74 for Type I and *r* = −0.66 for Type II SVs) and with both the arrival time and tethering lifetime of Type II SVs (Figure 2E and *r* = −0.69 for arrival times and *r* = −0.66 for tethering lifetime). The negative correlation between the straightness parameter and these times is consistent with Equation (1), which can be cast into

(2) $$S=\frac{\tau_{0}}{\tau }$$

where *τ* denotes one of the fusion time, arrival time, and tethering lifetime. *τ*_0_ denotes the value of *τ* in the hypothetical case where SV motion is purely ballistic, or where *S* = 1; *τ*_0_ can be estimated by *L*/*v_p_* with *v_p_* denoting the path‐averaged speed defined by *v_p_* ≡ Λ/*τ*. We found that the path‐averaged speed *v_p_* is nearly independent of the initial distance *L* (see Figure 2F and *r* = 0.21 for Type I SVs and *r* = 0.19 for Type II SVs) and has a similar mean value for both SV types (see Figure 2F, mean path‐averaged speed with 550 µm s^−1^ for Type I SVs and 538 µm s^−1^ for Type II SVs); consequently, the value of *τ*_0_ (= *L*/*v_p_*) is directly proportional to the initial distance *L* for both SV types. We found that Equation (2) provides a quantitative explanation of our experimental data for the straightness dependence of the fusion times of both Type I and Type II SVs (Figure 2D) as well as the straightness dependence of the arrival times and tethering lifetimes of Type II SVs (Figure 2E). The values of *τ*_0_ extracted from this analysis are found to be comparable to the *τ*_0_ values estimated from its definition, i.e., *L*/*v_p_* (**Table** **1**). We note that Equation (2) holds regardless of SV fusion dynamics. As discussed later in this work, the correlation between the straightness parameter and the initial distance has a significant effect on the SV fusion dynamics.

**Table 1:** Comparison between two differently measured ballistic times. τ_0_ represents the ballistic time scale identified from Equation (2), i.e., the time interval *τ* when the straightness parameter *S* equals unity. The value of *τ*_0_ was obtained from the best fit of Equation (2) to each data displayed in Figure 2D,E. 〈*L*/*v_p_* 〉 represents the mean ballistic time, where *L* and *v_p_* denote, respectively, the end‐to‐end distance and path‐averaged speed for a given SV trajectory in each dataset (see the text below Equation 2).

Dataset	*τ*_0_ [s]	〈*L*/*v_p_* 〉 [s]
Fusion time, Type I SVs	0.166	0.202
Fusion time, Type II SVs	0.475	0.587
Arrival time, Type II SVs	0.390	0.489
Tethering lifetime, Type II SVs	0.193	0.247

### 2.3 Direction‐Dependent Straightness and Mean Velocity of SV Motion upon Electrical Stimulation

**Figure 3:**
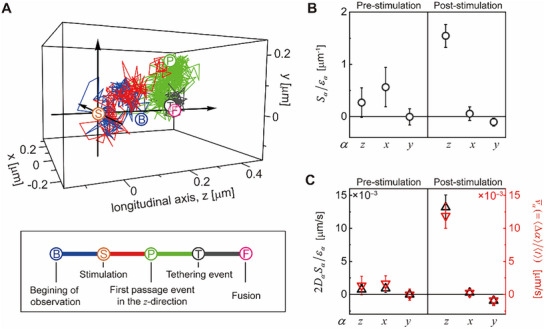
Mean straightness varies with the direction of SV motion and electrical stimulation. A) A representative SV trajectory. The blue, red, green, and dark gray lines represent, respectively, the pre‐stimulation, first passage, post‐first passage, and intra‐fusion domain segments of the SV trajectory. The longitudinal z‐axis is defined as the straight line connecting the stimulation (Ⓢ) and fusion (Ⓕ) sites, and the transverse *x*‐and *y*‐axes are the two mutually orthogonal axes perpendicular to the z‐axis. The first passage site (Ⓟ) denotes the position at which an SV first crosses the (*x*, *y*)‐plane boundary located at the tethering site (Ⓣ). B, C) Scaled mean straightness, *S*_*α*_/*ε*_*α*_and 2*D*_*α*_ *S*_*α*_/*ε*_*α*_, along the *α*‐axis (α ∈ {*x*, *y*, *z*}): (left and right) results obtained from Type II SV trajectories (*n* = 65) before and after stimulation. In B, C), the error bars represent the standard error of the mean. In C), the scaled mean straightness is compared with the mean velocity calculated as 〈Δ*α*〉/〈*t*〉, with 〈Δ*α*〉 and 〈*t*〉 denoting the mean displacement along the a‐axis and the mean of *t*. Upon stimulation, the scaled mean straightness in the longitudinal direction increases more than tenfold compared to its pre‐stimulation value, while the mean straightness in the transverse directions remains largely unchanged.

Dynamics of SV motion upon electrical stimulation are strongly dependent on the direction of motion. For each SV trajectory, we defined the longitudinal direction as the direction from the SV position at the onset of electrical stimulation to the SV fusion site, which is chosen as the direction of the *z*‐axis (see **Figure** **3**A). On the other hand, the two transverse directions orthogonal to the longitudinal direction are chosen to be the directions of the *x*‐and *y*‐axes. Upon electrical stimulation, the mean straightness parameter *S_z_* along the longitudinal direction drastically increases; on the other hand, the mean straightness parameters, *S_x_* and *S_y_*, along the transverse directions do not (Figure 3B). Here, the mean straightness *S*_*α*_ along the *α*‐axis is defined as *S*_*α*_ = 〈Δ*α*〉/〈*l*_*α*_〉 (*α* ∈ {*x*, *y*, *z*}) for a trajectory from (*x*_0_,*y*_0_,*z*_0_) to (*x*_1_,*y*_1_,*z*_1_), where Δ*α* and *l*_*α*_ denote the displacement, *α*_1_ − *α*_0_, along the *α*‐axis and the pathlength of the *α*‐axis projection of the trajectory (see Methods for more details).

The increase in *S_z_* signifies an increase in the mean force exerted on the SVs along the longitudinal direction. We found that, for a 1D random walk under a constant force field along the *α*‐axis, the mean straightness parameter, *S*_*α*_, of the random walker's trajectory is the same as the difference between the probability, *p*_*α*,+_, of the walker stepping in the positive direction and the probability, *p*_*α*,−_, of stepping in the negative direction (see Text S2, Supporting Information). This difference, *p*_*α*,+_ − *p*_*α*,−_, is well known to be directly proportional to the mean force *F*_*α*_ exerted on the random walker, i.e.,

(3) $$S_{\alpha }=p_{\alpha ,+}-p_{\alpha ,-}=F_{\alpha }\varepsilon_{\alpha }/\left(2k_{B}T\right)$$

where *ε*_*α*_, *k_B_* and *T* denote, respectively, the lattice constant along the *α*‐axis, Boltzmann's constant, and temperature. In the presence of an absorbing boundary on the lattice, Equation (3) holds if the mean force is directed toward the absorbing boundary. Otherwise, *S*_*α*_ vanishes (Text S2, Supporting Information). We note here that, for the random walk model, *S*_*α*_/*ε*_*α*_ depends only on the energetic properties such as the mean force and temperature but is independent of the initial position and the diffusion coefficient of the random walker. Equation (3) holds for a continuous‐time random walker model as well,^[^ ^28^, ^32^ ^]^ regardless of detailed form of the waiting time distribution, as long as the distribution has a finite first moment. In the continuum limit, a random walker undergoes the Brownian motion, for which the pathlength of any individual trajectory diverges, and the straightness parameter vanishes. However, *S*_*α*_/*ε*_*α*_ of a biased random walker converges to *F*_*α*_/(2*k_B_T*) in the continuum limit. In experiment, both *S*_*α*_ and *ε*_*α*_ depend on the experimental time resolution, but their ratio should approach the limiting value when the experimental time resolution is sufficiently high.

The mean velocity of SV increases with the mean straightness parameter and the diffusion coefficient. One can show that the mean velocity, ${\bar{v}}_{\alpha }$, defined by ${\bar{v}}_{\alpha }\equiv \left\langle \Delta \alpha \right\rangle /\left\langle t\right\rangle$, is related to *S*_*α*_ by

(4) $${\bar{v}}_{\alpha }=2D_{\alpha }S_{\alpha }/\varepsilon_{a}$$

where *D*_*α*_ denotes the diffusion coefficient defined by $\varepsilon_{\alpha }^{2}/2\Delta t$. We found that the mean velocity estimated based on Equation (4) is in good agreement with the direct experimental estimation of the mean velocity, 〈Δ*α*〉/〈*t*〉 with 〈*t*〉 denoting the mean lifetime of SV trajectories (Figure 3C). The mean velocity along the *z*‐axis estimated from Equation (4) is given by 7.38 × 10^−1^ nm s^−^^1^ before electrical stimulation but 1.32 × 10 nm s^−1^ after the stimulation, which are close to the mean velocity directly estimated by 〈Δ*α*〉/〈*t*〉, given by 1.34 nm s^−1^ before stimulation and 1.19 × 10 nm s^−1^ after the stimulation. Note that the mean SV velocity during the electrical stimulation is 17.9 folds higher than the mean velocity before the stimulation. The increase in the mean velocity primarily results from a rise in the straightness parameter along the longitudinal direction upon the electrical stimulation. In contrast, there is no significant electrical stimulation induced change in the mean velocity along *x*‐ and *y*‐axes. The diffusion coefficient *D*_*α*_ of SV motion along the transverse directions (*α* = *x* or *y*) is not much different from *D_z_*. The diffusion coefficients show little change in any direction upon electrical stimulation, either.

### 2.4 Longitudinal Motion of Type II SV during Electrical Stimulation: Positive Correlation between the Mean Force and Initial Distance

**Figure 4:**
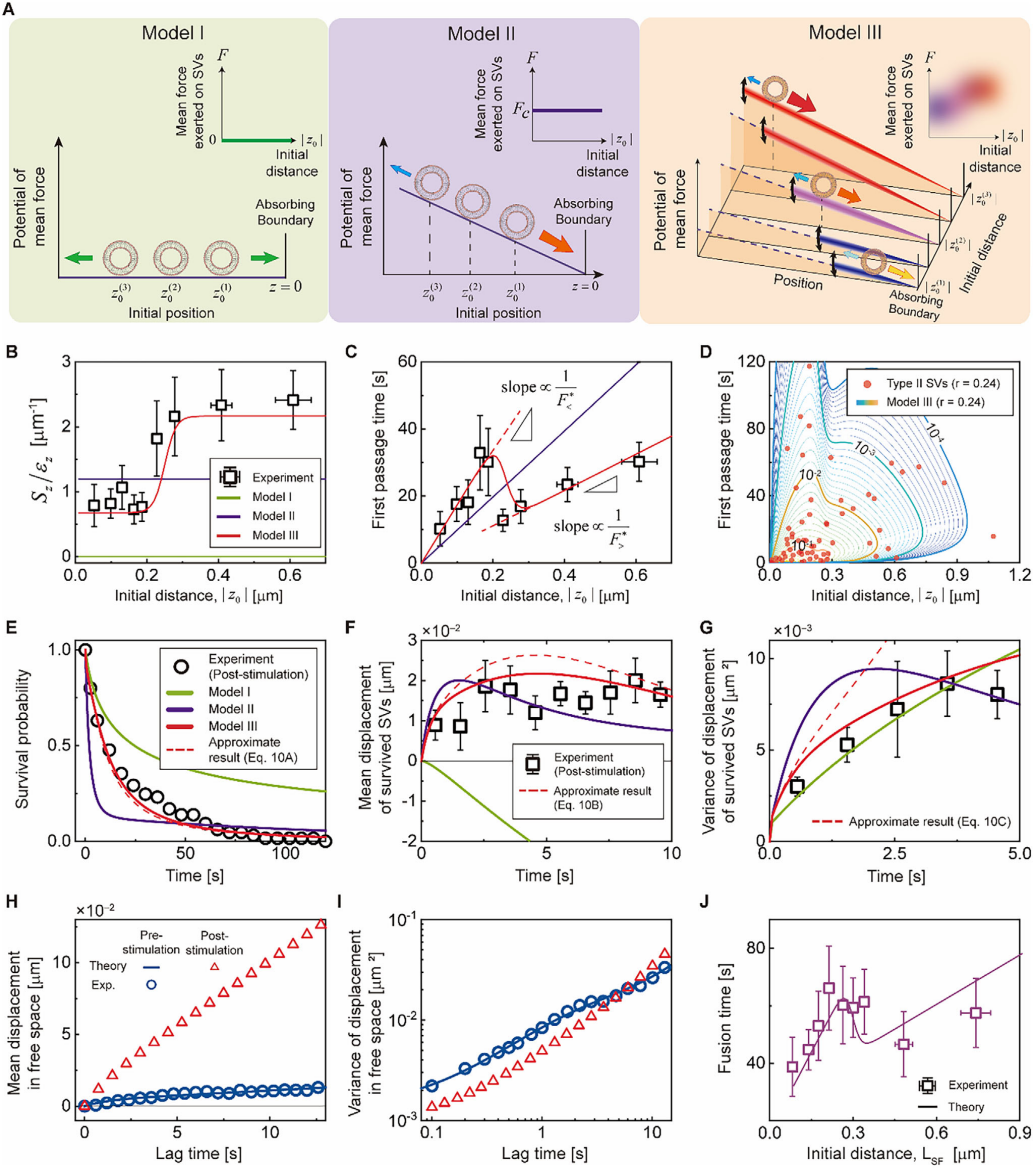
Quantitative analyses of longitudinal motion of Type II SVs. A) Schematics of three different models for the mean force acting on SVs. Model I assumes no external force. In Model II, SVs are subjected to a constant external force, *F_c_*. In Model III, SVs are subjected to distributed external forces that correlates with their initial distances from the absorbing boundary, where the initial distance, |*z*_0_|, corresponds to the distance between the stimulation site and the tethering site in the longitudinal direction. The inset in each panel describes how the mean force and mean straightness in the model depend on the initial distance. B,C) Initial distance dependence of the scaled mean straightness, *S_z_*/*ε*_*z*_, and the first passage time (*n* = 65). In B,C), the symbols represent the experimental data, calculated as the averages for each bin containing thirteen data points, with error bars representing standard error of the mean. D) Relationship between the initial distance and the first passage time. The contour lines represent the joint probability density of the initial distance and first passage time, obtained for Model III. E) Survival probability and F) and G) survival probability‐weighted mean and variance of the SV displacement. The symbols represent the experimental data; in F,G), the squares indicate averages for each 1‐s‐width bin, with error bars representing standard deviations. In B),C), and E‐G), the green, purple, and red solid lines represent the best‐fit results of Model I, II, and III, respectively (see Equations (30), (31), (32), (33), (34), (35),  39, and 41 in Methods). In E‐G), the red dashed lines represent the results of the approximate solution (Equation 10) using the best‐fit parameters of Model III. H) Mean and I) variance of the SV displacement in the absence of boundary (see Equations (31), (32), and (36) in Methods). The blue circles and lines represent, respectively, the experimental data and the best‐fit results of Model III before stimulation. The red triangles represent the theoretical predictions of the optimized Model III for the mean and variance of the SV displacement in the absence of boundary. A notable increase in slope along the longitudinal direction is observed in H) compared to the pre‐stimulation case, reflecting a corresponding increase in the mean force. J) Initial distance (*L*_SF_) dependence of the fusion time for Type II SVs (see also Figure 1H). The solid line represents the theoretical result for Model III (see Equation (45) in Methods).

Motion of Type II SVs during electrical stimulation in the presynaptic terminal has stochastic properties that cannot be explained by previously existing models of passive motion^[^ ^19^, ^33^ ^]^ or the recently reported model of active SV motion in the axon away from the presynaptic terminal.^[^ ^34^, ^35^ ^]^ We investigated the dependence of the straightness parameter and the first passage time of Type II SVs on the initial longitudinal distance from their tethering sites, as well as the time‐dependence of the survival probability, *P_S_* (*t*), and the survival probability weighted mean, Δ_1_(*t*), and variance, *σ* ^2^(*t*), of their longitudinal displacement (**Figure** **4**), which can be estimated by

(5) $$P_{S}\left(t\right)=N\left(t\right)/N\left(0\right)$$

(6) $$\Delta_{1}\left(t\right)=\frac{1}{N(0)}\sum_{i=1}^{N(t)}\Delta z_{i}\left(t\right)$$

(7) $$\sigma^{2}\left(t\right)=\frac{1}{N(0)}\sum_{i=1}^{N(t)}{\left(\Delta z_{i}\left(t\right)-\left\langle \Delta z\left(t\right)\right\rangle \right)}^{2}$$

where *N*(*t*), Δ*z_i_* (*t*) ≡ *z_i_* (*t*) − *z_i_* (0), and 〈Δ*z*(*t*)〉 denote, respectively, the number of SVs that have not undergone the first passage event as of time *t*, the displacement of the *i‐*th SV in the longitudinal direction over the time interval (0, *t*), and the trajectory average of the displacement Δ*z_i_* (*t*). These experimental data could not be explained by taking the conventional reaction‐diffusion equation or its modern generalization based on the continuous time random walker models.^[^ ^28^, ^36^, ^37^, ^38^ ^]^ To explain our experimental data, we introduced a model for SV transport in the presynaptic terminal. In this model, stochastic motion of SVs inside the crowded presynaptic terminal^[^ ^39^ ^]^ is modeled as a reversible switching process between unbound diffusion and bound diffusion (see Text S3, Supporting Information). The bound diffusion is modeled as the Ornstein‐Uhlenbeck process, which represents thermal motion of a particle under a harmonic potential.^[^ ^40^ ^]^

For our model, the transport equation governing the longitudinal SV motion at the presynaptic terminal is given by

(8) $$s\hat{P}\left(z,s|z_{0},F\right)-\delta \left(z-z_{0}\right)=\hat{D}\left(s\right)\frac{\partial }{\partial z}\left[\frac{\partial }{\partial z}-\beta F\right]\hat{P}\left(z,s|z_{0},F\right)$$

in the Laplace domain (see Methods). Throughout, $\hat{f}\left(s\right)$ designates the Laplace transform, i.e., $\hat{f}\left(s\right)=\int_{0}^{\infty }dte^{-st}f\left(t\right)$. *P*(*z*, *t*|*z*_0_,*F*) denotes the conditional probability of finding an SV at position *z* at time *t*, given that it is initially located at *z*_0_(< 0) under the constant mean force *F*. *δ*(*z*) and *β* denote Dirac's delta function and *β* = 1/(*k_B_T*), respectively. To obtain the first passage time distribution, we imposed the absorbing boundary condition, *P*(0, *t*|*z*_0_,*F*) = 0, at *z* = 0 which is chosen to be the longitudinal position of the tethering point (see Figure 3A). In Equation (8), $\hat{D}\left(s\right)$ denotes the Laplace transform of the diffusion kernel obtained for our model of SV motion, given by

(9) $$\hat{D}\left(s\right)={\left(s+\lambda \right)}^{-1}\left(sD_{s}+\lambda D_{\infty }\right)$$

where *D_s_* and *D*_∞_ denote the diffusion coefficient values at short times and at long times, respectively. In Equation (9), *λ* denotes the transition rate from short‐time to long‐time diffusion. The parameter *λ* is dependent on the transition rate between unbound and bound states as well as the relaxation time characterizing the SV motion in the bound state in our model (see Text S3 for more details, Supporting Information). Starting from Equations (8) and (9), we obtained analytic results in the Laplace domain for the first passage time distribution and the time‐dependent mean and variance of the displacement of SVs that have yet to undergo the first passage (see Methods).

Our experimental data could not be explained if we assumed no external force or constant external force *F* exerted on SVs. If there is no external force (Model I), the mean straightness parameter should vanish regardless of the initial distance (see Figure 4A and Methods). If there is a constant external force *F* (Model II), the mean straightness parameter should be constant independent of the initial distance (see Figure 4A and Methods). The predictions of these models are found to be inconsistent with our experimental results for the initial distance dependence of the mean straightness parameter (Figure 4B) and the mean first passage time (Figure 4C); not to mention, these models could not simultaneously explain the time‐dependence of the survival probability of Type II SVs and the mean and variance of their displacements, either (Figure 4D–G).

Motion of Type II SVs upon electrical stimulation could be quantitatively explained by assuming that the mean force, *F*, exerted on an SV is distributed and positively correlated with its initial distance, *z*_0_. Our experimental data showed that straightness parameter is distributed (Figure 2C) and its longitudinal component *S_z_* exhibits a sigmoidal dependence on the initial distance, |*z*_0_| (Figure 4B). According to this observation and Equation (3), we hypothesized that the mean force *F* is distributed and its expectation value increases with the initial distance |*z*_0_| in the sigmoidal manner (see Model III in Figure 4A and Equation (35) in Methods). For this model, called Model III, the analytic expressions of the experimental observables can be obtained from Equation (8) with Equation (9) (see Equations (30), (31), (32)), (35), (43), and (45 in Methods). We found that Model III provides a simultaneous, quantitative explanation of various experimental results, including the initial distance dependence of the mean straightness and the mean first passage time, the small Pearson correlation coefficient value of 0.24 between the first passage times and initial distances, and the three experimental observables defined in Equations ((5), (6), (7)) (Figure 4B–G). The optimized values of adjustable parameters in Model III are listed in **Table** **2**. Using the optimized Model III, we found the mean velocity of SVs to be ${\bar{v}}_{z}\left(=\beta \left\langle F\right\rangle D_{\infty }\right)≅12.8$ nm s^−1^ (see the caption of Table 2), which is also comparable to the direct experimental estimation, 11.9 nm s^−1^ (Figure 3C).

**Table 2:** Optimized parameter values obtained from our quantitative analysis for SV motion in the presynaptic terminal for each model. *D_s_*, *D*_∞_, and *λ *denote parameters associated with the diffusion kernel given in Equation (9), which describes a transition from the short‐time diffusion coefficient, *D_s_*, to the long‐time diffusion coefficient, *D*_∞_, at a rate of *λ*. ${\left\langle \delta z_{QS}^{2}\right\rangle }^{1/2}$ represents the magnitude of QD fluctuations centered at a given SV position (see Equation 39). *F_c_* is the mean force parameter uniformly applied to every SV in Model II, regardless of SV positions upon stimulation. *a_F_* and *b_F_* are shape and scale parameters characterizing the mean force distribution that is assumed to be a gamma distribution in Model III (see Equation 35); in the post‐stimulation case, *b_F_* is not a constant but a function of the initial distance, |*z*_0_|.

Parameter		Model I	Model II	Model III	
		Post‐stimulation	Post‐stimulation	Post‐stimulation	Pre‐stimulation
Common	*D_s_* [nm^2^ s^−1^]	1.56 × 10^3^	7.20 × 10^3^	4.45 × 10^3^	4.89 × 10^3^
	*D*_∞_ [nm^2^ s^−1^]	1.50 × 10^3^	1.05 × 10^3^	1.23 × 10^3^	8.07 × 10^2^
	*λ* [s^−1^]	2.58 × 10^−7^	5.81 × 10^−2^	2.30 × 10^0^	8.53 × 10^−1^
	${\left\langle \delta z_{QS}^{2}\right\rangle }^{1/2}$ [nm]	2.12 × 10^1^	1.87 × 10^1^	2.22 × 10^1^	2.59 × 10^1^
Model II specific	*F_c_* [pN]	‐	4.01 × 10^−2^	‐	‐
Model III specific	*a_F_*	‐	‐	4.26	2.31
	*b_F_* [pN]	‐	‐	*b_F_* = *b_F_* (*z*_0_)^a)^ *b*_<_ = 3.58 × 10^−4^ pN *b*_>_ = 1.15 × 10^−3^ pN *γ* = 3.25 × 10^−2^ nm^−1^ *l_c_* = 2.50 × 10^2^ nm	1.53×10^−3^

^a)^ Initial distance‐dependent scale factor *b_F_* (*z*_0_) is given by *b_F_* (*z*_0_)= *b*_<_ $+\left(b_{>}-b_{<}\right)/\left[1+e^{-2\gamma (|z_{0}|-l_{c})}\right]$, which undergoes a transition from *b*_<_ to *b*_>_(> *b*_<_ > 0) as |*z*_0_| increases with a slope (*b*_>_ − *b*_<_)*γ*/2 at |*z*_0_| = *l_c_*.

Our models yield exact analytic results in the Laplace transform domain for those experimental observables defined in Equations ((5), (6), (7)), i.e., the survival probability *P_S_* and the first and second moments of the survival probability weighted displacement distribution *P*(*z*, *t*|*z*_0_,*F*) (see Equations ((30), (31), (32)) in Methods). For Model II, the approximate time domain expressions for these experimental observables are obtained as

(10) $$P_{S}\left(t|z_{0},F\right)≅\Delta_{0}\left(t|z_{0},F\right)=C_{+}\left(t|z_{0},F\right)-e^{-\beta Fz_{0}}C_{-}\left(t|z_{0},F\right)$$

(11) $$\Delta_{1}\left(t|z_{0},F\right)≅\beta FD_{\infty }\tau \left(t\right)P_{S}\left(t|z_{0},F\right)+2z_{0}e^{-\beta Fz_{0}}C_{-}\left(t|z_{0},F\right)$$

(12) $$\begin{array}{c} \begin{array}{c} \Delta_{2}\left(t|z_{0},F\right)≅\left[2D_{\infty }\tau \left(t\right)+{\left[\beta FD_{\infty }\tau \left(t\right)\right]}^{2}\right]P_{S}\left(t|z_{0},F\right)\\ -4z_{0}\left[C_{-}\left(t|F,z_{0}\right)\left[z_{0}-\beta FD_{\infty }\tau \left(t\right)\right]+\sqrt{\frac{D_{\infty }\tau \left(t\right)}{4\pi }}e^{-{\left[z_{0}+\beta FD_{\infty }\tau \left(t\right)\right]}^{2}/4D_{\infty }\tau \left(t\right)}\right] \end{array} \end{array}$$

where *z*_0_ and $\Delta_{n}\left(t|z_{0},F\right)$, respectively, designate the initial position of an SV and the *n*‐th moment of the longitudinal displacement distribution, defined by $\Delta_{n}\left(t|z_{0},F\right)=\int_{-\infty }^{0}{\left(z-z_{0}\right)}^{n}P\left(z,t|z_{0},F\right)dz$. In Equation (10), *C*_±_(*t*|*z*_0_,*F*) and *τ*(*t*) are given by *C*_±_(*t*|*z*_0_,*F*) $=\frac{1}{2}\left[1\mp \mathrm{erf}(\left[z_{0}\pm \beta FD_{\infty }\tau \left(t\right)\right]/\sqrt{4D_{\infty }\tau \left(t\right)})\right]$ and $\tau \left(t\right)=t+\left(D_{s}/D_{\infty }-1\right)\left(1-e^{-\lambda t}\right)/\lambda$ with erf(*x*) denoting the error function (see Equations (46 and 47); Text S4, Supporting Information). For each of Equations ((10), (11), (12)), the corresponding equation for Model III can be obtained by taking average over the joint distribution *p*_III_(*z*_0_,*F*) of the initial distance and the mean force, given in Equation (35) in Methods. These approximate results are found to be comparable to the experimental results and the exact theoretical results, obtained from the numerical inverse Laplace transform of Equations ((30), (31), (32)) in Methods (Figure 4E–G).

### 2.5 Longitudinal Motion of Type II SVs before Electrical Stimulation

Before electrical stimulation, Type II SVs have far smaller mean velocity in the longitudinal direction than during stimulation. We found that the mean and variance of the Type II SV displacement before electrical stimulation can also be explained by the solution of Equations (8) and (9) (see Table 2 for the extracted parameter values). The extracted value of mean force *F* before stimulation is found to be about twelve times smaller than that during stimulation. On the other hand, the short‐ and long‐time diffusion coefficients of SVs before stimulation have similar values to those during electrical stimulation.

Even before stimulation, the mean displacement of the Type II SVs toward fusion sites does not linearly increase with time: it increases faster at short times than at long times. This is because, for Type II SVs undergoing reversible trapping in a crowded environment, the short‐time diffusion coefficient is greater than the long‐time diffusion coefficient. Starting from Equations (8) and (9), we obtained analytic expressions for the mean and variance of the SV displacement in the absence of boundary for Model III (see Methods). The short‐time and long‐time asymptotic behaviors of the mean displacement are given by

(13) $$\Delta_{1}\left(t\right)=\left\{\begin{array}{cc} \beta \left\langle F\right\rangle D_{s}t & \;\lambda t<<1\\ \beta \left\langle F\right\rangle D_{\infty }t & \;\lambda t>>1 \end{array}\right.$$

This transition in the mean SV displacement occurs regardless of fluctuation in external force *F*. On the other hand, the variance of the SV displacement in the absence of any boundary is given by

(14) $$\begin{array}{c} \Delta_{2}\left(t\right)-\Delta_{1}^{2}\left(t\right)=\left\{\begin{array}{cc} 2D_{s}t & \;\lambda t<<1\\ \begin{array}{c} 2D_{\infty }t+\beta^{2}\left\langle \delta F^{2}\right\rangle D_{\infty }^{2}t^{2}\;+2\beta^{2}[\left\langle \delta F^{2}\right\rangle \\ +\left\langle F^{2}\right\rangle ]D_{\infty }\left(D_{s}-D_{\infty }\right)t/\lambda \end{array} & \;\lambda t>>1 \end{array}\right. \end{array}$$

The variance in the SV displacement exhibits different dynamic behavior depending on the fluctuation of *F*. The short‐time asymptotic behavior of the variance is not sensitive to external force fluctuation. However, at long times, the variance in the SV displacement becomes a super‐linear, quadratic function of time with the coefficient of the quadratic term proportional to the force fluctuation. The transitions from short‐time to long‐time asymptotic behaviors in the mean and variance of the SV displacement, given in Equations (13) and (14), are found to be consistent with our experimental results (Figure 4H,I).

Our quantitative analysis of the SV displacement before stimulation shows that the long‐time diffusion coefficient *D*_∞_ (≅ 8.07 × 10^2^ nm^2^ *s* ^−^) is indeed smaller than the short‐time diffusion coefficient *D_s_* (≅ 4.89 × 10^3^ nm^2^ *s* ^−1^) (Figure 4G,H), both of which are similar to the corresponding diffusion coefficients during electrical stimulation, i.e., *D*_∞_ (≅ 1.23 × 10^3^ nm^2^ *s* ^−1^) and *D_s_* (≅ 4.45 × 10^3^ nm^2^ *s* ^−1^). These results are consistent with our hypothesis that the Type II SVs undergo reversible trapping by proteins in the crowded cell environment in the presynaptic terminal of neurons, which causes the long‐time diffusion coefficient to be smaller than the short‐time diffusion coefficient.

### 2.6 Initial Distance Dependence of Fusion Time

Model III, in which we assume the sigmoidal dependence of the mean force on the initial distance, quantitatively explains the non‐monotonic dependence of the mean first passage time of SVs on the initial distance |*z*_0_| between the stimulation site and the tethering site in the longitudinal direction (Figure 4C). The fusion time of Type II SVs exhibits a similar non‐monotonic dependence on their initial distance *L*_SF_ between the stimulation site and the fusion site as observed in the dependence of the longitudinal first passage time on the initial distance |*z*_0_| (Figures 1H and 4J). We could quantitatively understand the experimental data for the non‐monotonic *L*_SF_ dependence of the fusion time, by noting that the fusion time *t_F_* of Type II SVs comprises the longitudinal first passage time, *t*_*FP*,*z*_, and the remaining time, *t_R_* (≡ *t_F_* − *t*_*FP*,*z*_), leading up to the final fusion event. The remaining time *t_R_* is found to have a negligible correlation with *L*_SF_ compared to the longitudinal first passage time *t*_*FP*,*z*_ (Figure 4C; Figure S3, Supporting Information); in addition, the distance *L_SF_* is found to be linearly related to |*z*_0_| (Figure S4, Supporting Information), i.e., *L*_SF_ = *A*|*z*_0_| + *B*, with *A* = 0.943 and *B* = 7.18 × 10^−2^ µm. Based on these observations, we could obtain a relationship of the mean fusion time τ_*F*_ (= 〈*t_F_* 〉) and *L*_SF_ by τ_*F*_ = 〈*t*_*FP*,*z*_(*z*_0_)〉 + 〈*t_R_* 〉 with |*z*_0_| = (*L*_SF_ − *B*)/*A*. Using the latter equation with 〈*t*_*FP*,*z*_(*z*_0_)〉 calculated by Model III, we could explain the non‐monotonic dependence of the fusion time on the distance between the stimulation point and the fusion sites (Figure 4J). This result demonstrates that the non‐monotonic *L*_SF_ dependence of fusion time originates mainly from that of the first passage time.

## 3 Discussion

Our analyses show that electrical stimulation increases the mean straightness of the Type II SV trajectories by more than tenfold in the longitudinal direction, leading to an order‐of‐magnitude increase in the mean velocity of Type II SVs toward their fusion sites (Figure 3C). The mean straightness parameter of SV trajectories introduced in this work is directly proportional to the mean force scaled by thermal energy but independent of the diffusion coefficient. Interestingly, SVs initially located farther from their tethering sites tend to have higher mean velocity during stimulation, because the straightness of their trajectories, or the mean force exerted on SVs, increases with their initial distance during stimulation (Figure 4B). These findings clearly show that the SV transport dynamics depends not only on electrical stimulation but also on the initial distance from the fusion domain at the onset of stimulation.

Type I and II SVs exhibit significantly different fusion time distributions and play different roles in synaptic transmission. The fusion times of Type I SVs follow a quasi‐exponential distribution with a mean of 21.7 s. On the other hand, Type II SVs have a far broader, non‐exponential fusion time distribution with a mean of 51.7 s. This discrepancy arises because the fusion process of Type II SVs is significantly contributed from the SV's biased motion from their initial positions to tethering sites, where their arrival times have a broad distribution. The broad fusion time distribution of Type II SVs plays critical roles in sustaining neurotransmission without fatigue during prolonged stimulation, which effectively supplies SVs containing neurotransmitters for a broad range of times after fast neurotransmitter release by Type I SVs.

Type I SVs exhibit confined motion near fusion sites prior to fusion and undergo fusion at early time points during electrical field stimulation. These SVs are available for fast release during stimulation and play a crucial role in fast synaptic transmissions.^[^ ^41^ ^]^ On the other hand, Type II SVs undergo unconfined motion toward their fusion sites during electrical stimulation and undergo fusion at late time points during stimulation. Type II SVs belong to the recycling pool, which replenishes the readily releasable pool (RRP) when the SVs in the RRP become depleted during electrical stimulation.^[^ ^42^, ^43^ ^]^ Our quantitative analyses clearly show that the biased motion of the Type II SVs during electrical stimulation results primarily from biased force exerted on SVs toward the fusion domain rather than unbiased thermal motion.

The presence of a mean force exerted on SVs toward the fusion domain is supported by the observation of a positive mean SV displacement (Figure 4F). In the absence of the mean force, the mean SV displacement becomes negative, as demonstrated by Model I. This occurs because the depletion of the probability distribution near the absorbing boundary shifts the most probable position away from the boundary, resulting in a net negative displacement.

This sigmoidal dependence of the mean force is consistent with our experimental data for the initial distance dependence of the scaled mean straightness in Figure 4B. The sigmoidal dependence of the mean force exerted on SVs with respect to their initial distances gives rise to the non‐monotonic dependence of both the mean first passage time and the fusion time of SVs on the initial distance. As shown in Figure 4C, the mean first passage time linearly increases with the initial distance, but with different slopes before and after approximately 200 nm. It is well established that, for a Brownian particle under the presence of a constant force toward a boundary, the mean first passage time linearly increases with its initial distance from the boundary, with the slope inversely proportional to the magnitude of the force.^[^ ^27^ ^]^ Therefore, the decrease in the slope near the critical initial distance, approximately 200 nm, suggests a transition from a small to a large force at the critical initial distance. When the force is distributed according to a gamma distribution at a given initial distance, the mean first passage time is inversely proportional to the most probable value of the force distribution at the distance (Equation 43). The greater mean force on SVs with larger initial distances from the fusion sites would facilitate the replenishment of the depleted SVs from the RRP, ensuring an efficient supply of SVs.^[^ ^44^ ^]^

Assuming a heterogeneity in the mean force is essentially required for the simultaneous quantitative explanation of various experimental results shown in Figure 4. In Model III, the distribution of the mean force is modeled as a gamma distribution whose expectation value is dependent on the initial distance in the sigmoidal manner. The heterogeneity in the mean force causes the SV fusion time to have a far broader distribution compared to the fusion time distribution of the hypothetical case with a constant mean force, as assumed in Model II (Figure S5, Supporting Information). On the other hand, the sigmoidal dependence of the expectation value of the mean force reduces the mean first passage time to 21.3 s, about half the value, 36.4 s, calculated for the case where the mean force is distributed according to a gamma distribution without any dependence on the initial distance. Likewise, the sigmoidal dependence of the expectation value of the mean force reduces the variance of the first passage time by a third, leading to a slight increase in the relative variance.

The mechanisms underlying the sigmoidal initial‐distance dependence of the mean force and straightness parameter remain unclear. However, we suspect that, upon stimulation, the delivery of distant Type II SVs toward their fusion sites is facilitated by molecular motors including myosin,^[^ ^45^ ^]^ which could result in the sigmoidal dependence of the straightness parameter or the mean force on the initial distance. This initial distance dependence may be linked to the fact that the actin nanostructure varies with the distance from the active zone in the presynaptic terminal, which has been recently revealed by super‐resolution microscopy.^[^ ^46^ ^]^ Molecular motors traveling along linear actin filaments between the active zone and distant regions could contribute to a significant mean longitudinal force. In contrast, near the active zone, actin filaments form a meshwork with randomized orientations,^[^ ^46^ ^]^ which could hinder directed transport and thereby reduce the mean longitudinal force, or the corresponding straightness parameter. We leave further investigation into the microscopic mechanisms underlying the initial distance dependent straightness parameter for future research.

The mean straightness parameter introduced in this work can serve as a measure of the mean force exerted on SVs. As demonstrated in this work, three different models (Model I–III) have qualitatively different dependences of the mean straightness parameter on the initial distance, which enables us to identify an accurate model of the Type II SV motion in the presynaptic terminal. The straightness parameter introduced in this work can be also used to quantify the straightness of various types of intracellular organelle trajectories, ranging from trajectories of SV motion in inhibitory neurons^[^ ^6^, ^9^ ^]^ and in vulnerable neurons of neurodegenerative diseases^[^ ^12^ ^]^ to trajectories of mitochondria^[^ ^47^, ^48^, ^49^ ^]^ and lysosomes.^[^ ^50^ ^]^

Remarkably, we made direct observation of detethering events of tethered SVs. Those SVs approach the fusion domain, transiently remain in the domain, and move away from the fusion domain before they return to the fusion domain and undergo the final exocytosis. These detethering events of SVs are reminiscent of the previously reported depriming event of SVs.^[^ ^25^, ^41^ ^]^ This untethering event may represent bouncing back of SVs from the fusion domain, possibly caused by a mismatch between the priming components between SVs and the plasma membrane. The detailed mechanisms underlying the detethering of SVs require further investigation.

Recently, liquid–liquid phase separation of synaptic proteins in the presynaptic terminal has been reported to play an important role in the organization of SVs in the presynaptic terminal.^[^ ^51^, ^52^, ^53^, ^54^ ^]^ Particularly, synapsins were reported to cluster SVs and form condensates in the presynaptic terminal through liquid–liquid phase separation,^[^ ^51^ ^]^ which can influence the motion and dynamics of SVs. Moreover, condensates of synapsin were reported to mediate actin polymerization.^[^ ^55^ ^]^ Thus, further investigation is needed to understand how liquid–liquid phase separation of synaptic proteins influences the motion of SVs in the presynaptic terminal.

## 4 Conclusion

In conclusion, we quantitatively investigated the transport and exocytosis dynamics of individual SVs in hippocampal neurons during electrical stimulation, categorizing them into Type I, 46% of the observed SVs exhibiting only confined motion near fusion sites during stimulation before exocytosis, and Type II, the remaining 54% undergoing unconfined motion toward their fusion sites from a distance during stimulation, followed by the confined motion near fusion sites prior to the final exocytosis. The fusion time of Type I SVs follows a quasi‐exponential distribution with the mean of 21.7 s while the fusion time of Type II SVs follows a far broader non‐exponential distribution with the mean of 51.7 s, which originates from both the heterogeneously biased motion of these SVs and a distributed initial distance from their fusion sites. Electrical stimulation increases the straightness parameter of the Type II SV trajectories toward their fusion sites by an order of magnitude. The mean straightness parameter is linearly proportional to the mean force exerted on SVs and hence their mean velocity. The mean straightness parameter, or the mean force, increases in a sigmoidal manner with the initial distances of Type II SVs from their fusion sites at the onset of electrical stimulation. This leads to a counterintuitive, non‐monotonic dependence of the fusion time on the initial distance, and a weak correlation between the fusion times and the initial distances. We presented a model of the SV transport and fusion dynamics in the presynaptic terminal, providing a simultaneous, quantitative explanation of various experimental results regarding the SV dynamics before and after neuronal stimulation. This work reveals how distinct types of SV motion contribute to synaptic transmission.

## 5 Experimental Section

### 3D Tracking of SVs in Living Neurons

Real‐time imaging experiments were performed as described previously.^[^ ^22^ ^]^ A microscope (Ti‐S, Nikon) containing an oil immersion 100x apochromat objective (Nikon), custom‐built dual‐focus imaging optics and an electron multiplying (EM) CCD camera (Ixon+, Andor) were used to image fluorescence of quantum dots (QDs). The size of one pixel in the *x*‐*y* plane in the EM CCD camera was 160 nm. The dual‐focus imaging optics include a beamsplitter (Beamexpander, USA), a right‐ angle mirror, lenses and mirrors. The dual‐focus imaging optics divided the fluorescence signals of QDs into two beam pathways through the beamsplitter. Two fluorescence signals in two beam pathways were placed side‐by‐side in the camera chip using the right‐angle mirror and mirrors. Lenses in each beam pathway were positioned slightly differently to generate two different focal planes. A PIFOC objective scanner with long‐travel range (Physik Instrumente (PI)) was used to generate the empirically determined calibration curve. Custom‐built IDL coded programs were used to fit a 2D Gaussian function to the fluorescence images of QDs to locate a 2D centroid in the *x*‐*y* plane and to estimate the fluorescence peak intensities of QDs at two different focal planes (*I*_1_ and *I*_2_). Programs written in LabVIEW (National Instruments) were utilized to control a piezo actuator and acquire the z‐position to generate the calibration curve. The calibration curve showed the z‐position dependence of the relative peak intensity difference ((*I*_1_−*I*_2_)/(*I*_1_+*I*_2_)), which is used to calculate the position along the *z*‐axis. The positions of a QD along the *z*‐axis were estimated by calculating relative peak intensity difference ((*I*_1_−*I*_2_)/(*I*_1_+*I*_2_)) first and calculating the z‐position using the calibration curve. A 405 nm laser (CrystaLaser, USA) was used to illuminate QDs. In order to capture the fluorescence signals of QDs, a dichroic mirror (z405rdc, Chroma) and an emission filter (ET605/70m, Chroma) were used. The full‐width at half maximum (2.33 *σ*) as localization accuracy was ≈ 20 nm for *x*‐ and *y*‐localization and ≈ 30 nm for *z*‐localization in 10 Hz imaging.

### Real‐Time Imaging of Single Quantum Dots in Cultured Hippocampal Neurons

Primary hippocampal neurons were collected from CA3 and CA1 regions of postnatal day 0 (P0) rats (Sprague‐Dawley) pups and were cultured as previously described^[^ ^56^ ^]^ regardless of sex. All procedures were performed following the animal ethics committee (AEC)‐approved animal protocols including AEP‐2021‐0092 and AEP‐2024‐010 at the Hong Kong University of Science and Technology. Real‐time imaging experiments in living neurons were performed between 14 and 21 days in vitro (DIV). Streptavidin‐coated QDs (A10196, Life Technologies) were preincubated at room temperature for ≈ 1 h with biotinylated antibodies (105 311BT, Synaptic System) binding to the luminal domain of synaptotagmin 1 at a 1:1 ratio before the addition of the conjugates solution to a sample chamber. Since synaptotagmin 1 is reported to exist in excitatory and inhibitory SVs, the loading SVs with QDs conjugated antibodies against the luminal domain of synaptotagmin 1 can label both excitatory and inhibitory SVs. In order to increase the photostability of QDs and to prevent nonspecific binding, Dithiothreitol (DDT) (2.5 mm, D9779, Sigma‐Aldrich) and casein (0.04 mg ml^−1^, C3400, Sigma–Aldrich) were added to preincubation solution. Approximately 0.1 nm of antibody‐conjugated QDs was used in order to label less than one QD per bouton (please see the representative image of antibody‐conjugated QDs in boutons labeled with FM 4–64 in Figure S6, Supporting Information). The emission filter (HQ665LP, Chroma) was used to capture the fluorescence signal FM 4–64 (T3166, Life Technologies). Following the addition of preincubation solution to the sample chamber, neurons in the sample chamber were stimulated with custom‐built platinum electrodes connected to the Grass Stimulator (SD9, Grass Technologies) at 10 Hz for 120 s. After stimulation, neurons were incubated for 60 s without stimulation. Then, the sample chamber was washed for 20 min using a perfusion system. Approximately, 1 µm trypan blue (T8154, Sigma–Aldrich) was applied right before imaging to avoid effects of trypan blue. The switching time of solution was approximately 5 s. The imaging protocol consisted of 20 s without stimulation, then 120 s of 10 Hz stimulation, and a final 20 s without stimulation. The camera, laser, and stimulator were synchronized by a triggering signal from the camera, which drives a Digidata 1322A (Molecular Devices). Fluorescence images were taken through a frame‐transfer mode at 10 Hz with exposure time of 100 ms using an EMCCD camera with EM gain of 250. QDs inside SVs undergoing exocytosis immediately after the onset of stimulation exhibited weak fluorescence signals during the exposure time, making it difficult to locate the centroid of QDs accurately; consequently, these traces were excluded from the data analyses. In order to generate the stimulation protocol, the Clampex program (Molecular Devices) was used. Neurons were perfused with artificial cerebrospinal fluid (ACSF) solution (150 mm NaCl, 4 mm KCl, 2 mm CaCl_2_, 2 mm MgCl_2_, 10 mm Glucose, and 10 mm 4‐(2‐hydroxyethyl)‐1‐piperazineethanesulfonic acid (HEPES) (300–310 mOsm, pH 7.3 with NaOH)). dioxo‐6‐nitro‐7‐sulfamoyl‐benzo [f]quinoxaline (NBQX) (5 µm, Ascent Scientific, USA) and Amino‐5‐phosphonopentanoic acid (D‐APV (50 µm, Ascent Scientific) were added to ACSF solutions to prevent recurrent activity and synaptic plasticity. Exocytosis was identified by the observation of quenching (a sudden and irreversible fluorescence drop close to zero intensity)(please see the representative fluorescence trace in Figure S7, Supporting Information). Since quenching of QDs with trypan blue is an irreversible process, one experiment was performed using one coverslip. The presence of QDs inside SVs did not affect exocytosis and mobility of SVs in living neurons.^[^ ^22^ ^]^

### Statistical Analysis

Statistical analyses were performed using MATLAB (R2023a, The MathWorks, Inc.). For each SV trajectory, using its own path‐averaged speed and the standard deviation of SV speeds, data points were identified as outliers when both the SV speeds immediately before and after the point had absolute *z*‐scores exceeding three, and such noisy points were excluded. Data in Figure 4F,G are presented as mean ± standard deviation, whereas data in all other cases are presented as mean ± standard error of the mean. A *p* value < 0.05, determined using a two‐sided KS test (*kstest2* function in MATLAB), was considered statistically significant.

### Methods

*Relationship between mean straightness parameter and mean force*: To derive Equation (3) based on CTRW model, we considered a random walker on a 1D lattice along the *α*‐axis with a lattice space spacing, ε_α_. We can assume a random walker, initially located at *m* = 0, is found at *m* = *m*_*α*_ after *N*_*α*_ jumps with *m* being the site index. Then, the displacement, Δ*α*, and the pathlength, *l*_*α*_, are obtained by Δ*α* = *m*_*α*_ *ε*_*α*_ and *l*_*α*_ = *N*_*α*_ *ε*_*α*_, respectively. We define the mean straightness parameter *S*_*α*_ as *S*_*α*_ = 〈Δ*α*〉/〈*l*_*α*_〉 = 〈*m*_*α*_〉/〈*N*_*α*_〉.

The mean jump number along the *α*‐axis 〈*N*_*α*_〉 can be calculated using the waiting time distribution, *ψ*_*α*_(*t*), for a random walker's jump to one of two nearest neighboring sites in *α*‐axis. The mean jump number 〈*N*_*α*_(*t*)〉 at time *t* as $\left\langle {\hat{N}}_{\alpha }\left(s\right)\right\rangle =s^{-1}{\hat{\psi }}_{\alpha }\left(s\right)/\left[1-{\hat{\psi }}_{\alpha }\left(s\right)\right]$ in Laplace domain. For a waiting time distribution whose first‐order moment, 〈*τ*_*α*_〉, is finite, 〈*N*_α_(*t*)〉 becomes linear in time at long time, i.e., $\left\langle N_{\alpha }\left(t\right)\right\rangle ∼t/\left\langle \tau_{\alpha }\right\rangle$. When the lengths of trajectories *t* are heterogeneous but have a finite mean 〈*t*〉, the mean jump number 〈*N*_*α*_〉 is obtained by 〈*N*_*α*_〉 = 〈*t*〉/〈*τ*_*α*_〉. The mean displacement along the *α*‐axis *ε*_*α*_〈*m*_*α*_〉 can be calculated from the probability, *p*_*α*_(*m*,*t*), that a random walker is located at the *m*‐th site over an infinite 1D lattice at time *t*, which is given by^[^ ^57^, ^58^ ^]^

(15) $$p_{\alpha }\left(m,t\right)=\sum_{N=|m|}^{\infty }p_{\alpha }\left(m|N\right)P_{\alpha ,N}\left(t\right)$$

where *p*_*α*_(*m*|*N*) denotes the conditional probability that the random walker is located at the *m*‐th site, given that it has undergone *N* jumps and *P*_*α*,*N*_(*t*) denotes the probability that the total number of jumps made by a random walker is *N* at time *t* along the *α*‐axis (Text S2, Supporting Information).^[^ ^59^ ^]^ Performing the summation over *N* on the right‐hand side of Equation (15) in the Laplace domain, Equation (15) can be rewritten as

(16) $$\begin{array}{ccc} {\hat{p}}_{\alpha }\left(m,s\right) & = & \frac{1-{\hat{\psi }}_{\alpha }\left(s\right)}{s}\frac{{\left[\left[1+\mathrm{sgn}\left(m\right)2\delta_{\alpha }\right]\hat{h}\left(s\right)\right]}^{\left|m\right|}}{\sqrt{1-4p_{\alpha ,+}p_{\alpha ,-}{\hat{\psi }}_{\alpha }{\left(s\right)}^{2}}};\\ \hat{h}\left(s\right) & = & \frac{{\hat{\psi }}_{\alpha }\left(s\right)}{1+\sqrt{1-4p_{\alpha ,+}p_{\alpha ,-}{\hat{\psi }}_{\alpha }{\left(s\right)}^{2}}} \end{array}$$

where *p*_*α*,+(−)_ indicates the probability that the random walker makes a jump in the positive (negative) direction along *α*‐axis, here, which is explicitly given by *p*_*α*,±_ = 1/2 ± *δ*_*α*_ (0 ≤ *δ* ≤ 1/2) with *δ*_*α*_ being a parameter yielding a bias toward the positive direction of the *α*‐axis and sgn(*m*) denotes the sign of *m*. Using Equation (16), the Laplace‐domain expression of the mean displacement can be obtained as

(17) $$\varepsilon_{\alpha }\left\langle {\hat{m}}_{\alpha }\left(s\right)\right\rangle =\varepsilon_{\alpha }\sum_{m=-\infty }^{\infty }m{\hat{p}}_{\alpha }\left(m,s\right)$$

which reduces to $\varepsilon_{\alpha }\left\langle {\hat{m}}_{\alpha }\left(s\right)\right\rangle ≅2\delta_{\alpha }\varepsilon_{\alpha }/\left\langle \tau_{\alpha }\right\rangle s^{2}$ in the small‐*s* limit. That is to say, the mean displacement linearly increases with time at long times:

(18) $$\varepsilon_{\alpha }\left\langle m_{\alpha }\left(t\right)\right\rangle ≅\frac{\left(2\delta_{\alpha }\right)\varepsilon_{\alpha }}{\left\langle \tau_{\alpha }\right\rangle }t$$

Considering the heterogeneity in the lengths of trajectories, 〈*m*_*α*_〉 becomes 〈*m*_*α*_〉 = 2*δ*_*α*_〈*t*〉/〈*τ*_*α*_〉. We obtained the mean straightness, *S*_*α*_, as

(19) $$S_{\alpha }=\left\langle m_{\alpha }\right\rangle /\left\langle N_{\alpha }\right\rangle =2\delta_{\alpha }$$

It is known that the magnitude, *F*, of the gradient of the potential of mean force is related to *p*_*α*,±_ as *β* *F*_*α*_ = 2(*p*_*α*,+_ − *p*_*α*,−_)/*ε*_*α*_ with *β* = 1/*k_B_T*.^[^ ^39^, ^40^ ^]^ *k_B_* and *T* denote the Boltzmann constant and the absolute temperature, respectively; the associated potential of mean force is given by *U*(*α*) = −*F*_*α*_ *α*. For a constant bias, *S*_*α*_ can be expressed in terms of *F*_*α*_ as

(20) $$S_{\alpha }=\frac{\beta F_{\alpha }\varepsilon_{\alpha }}{2}$$

Second, let *p*_*α*_(*m*,*t*|*L*) denote the probability that a random walker is located at the *m*‐th site at time *t* under the absorbing boundary condition at *m* = *m_L_*, i.e., *p*_*α*_(*m_L_*,*t*|*L*) = 0. *p*_*α*_(*m*,*t*|*L*) or ${\hat{p}}_{\alpha }\left(m,s|L\right)$ can be obtained as

(21)

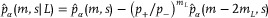

by using the conventional image method.^[^ ^27^ ^]^ The Laplace transform, ${\hat{P}}_{S,\alpha }\left(s|L\right)$, of the survival probability, *P*_*S*,*α*_(*t*|*L*), that a random walker does not reach the absorbing boundary by time *t* is then obtained by summing Equation (21) over *m*, which is given by

(22) $${\hat{P}}_{S,\alpha }\left(s|L\right)=\sum_{m=-\infty }^{m_{L}}{\hat{p}}_{\alpha }\left(m,s|L\right)$$

By taking the small‐*s* limit of Equation (22), we can obtain the mean first passage time, *τ*_*FP*,*α*_(*L*), as $\tau_{FP,\alpha }\left(L\right)={\hat{P}}_{S,\alpha }\left(0|L\right)=\left(m_{L}/2\delta_{\alpha }\right)\left\langle \tau_{\alpha }\right\rangle$. From the mean jump number at long times 〈*N*_*α*_〉_*L*_ = *τ*_*FP*,*α*_(*L*)/〈*τ*_*α*_〉 and a heterogeneity in the initial distance from the absorbing boundary with a finite mean 〈*m_L_* 〉, we obtained 〈*N*_*α*_〉 = 〈*m_L_* 〉/2*δ*_*α*_. Using the mean displacement *ε*_*α*_〈*m_L_* 〉 from the trajectories during the first passage event, we reproduced the same relationship between mean straightness and mean force as given in Equation (20).

The mean straightness, *S*_*α*_, shown in Figures 3 and 4, was calculated by *S*_*α*_ = 〈Δ*α*〉/〈*l*_*α*_〉 (*α* ∈ {*x*, *y*, *z*}) for a trajectory from (*x*_0_,*y*_0_,*z*_0_) to (*x*_1_,*y*_1_,*z*_1_), where Δ*α* and *l*_*α*_ denote the displacement, *α*_1_ − *α*_0_, along the *α*‐axis and the pathlength of the *α*‐axis projection of the trajectory. We used the 20‐s trajectories for the pre‐stimulation and the trajectories from the stimulation site (Ⓢ) to the first passage site (Ⓟ) for the post‐stimulation (Figure 3A). Here, *ε*_*α*_ and *D*_*α*_ denote, respectively, the mean jump length, 〈*l*_*α*_/*t*〉Δ*t*, during the experimental time resolution Δ*t* and the diffusion coefficient, $\varepsilon_{\alpha }^{2}/2\Delta t$, along the *α‐*axis. Here, *t* represents 20 s in the pre‐stimulation case and the first passage time for a given SV trajectory in the post‐stimulation case.

*Model of SV motion in presynaptic terminal*: To describe the motion of SVs in presynaptic terminals, we considered the generalized transport equation along the longitudinal *z*‐axis, which can be obtained using the projection operator technique as follows:^[^ ^60^ ^]^

(23) $$\begin{array}{c} \partial_{t}P\left(z,t|z_{0}\right)=\int_{0}^{t}dt^{\prime }\int dz^{\prime }\frac{\partial }{\partial z}D\left(z,z^{\prime },t^{\prime }\right)\left[\frac{\partial }{\partial z^{\prime }}-\beta F\left(z^{\prime }\right)\right]P\left(z^{\prime },t-t^{\prime }|z_{0}\right) \end{array}$$

where $D(z,z^{\prime },t)$ denotes the position‐dependent diffusion kernel, which effectively accounts for the retarded hydrodynamic interaction, or the influence of the flux at *z*′ at time zero to the flux at *z* at time *t*. In Equation (23), *F*(*z*) represents the mean force exerted on an SV at position *z*. In the hydrodynamic limit, where the length scale of the hydrodynamic interaction becomes negligible, the diffusion kernel can be written as $D\left(z,z^{\prime },t\right)=\delta \left(z-z^{\prime }\right)D\left(z^{\prime },t\right)$. In this case, Equation (23) reduces to

(24) $$\partial_{t}P\left(z,t|z_{0}\right)=\int_{0}^{t}dt^{\prime }\frac{\partial }{\partial z}D\left(z,t^{\prime }\right)\frac{\partial }{\partial z}\left[\frac{\partial }{\partial z}-\beta F\left(z\right)\right]P\left(z,t-t^{\prime }|z_{0}\right)$$

Assuming the environmental influence on $D(z,t^{\prime })$ is spatially homogeneous, i.e., $D\left(z,t^{\prime }\right)=D\left(t^{\prime }\right)$, and treating the mean force, *F*(*z*), as a *z*‐independent random variable, *F*, which allows different SVs to experience different forces, we obtained Equation (8) in the Laplace domain.

To complete our SV transport model, an explicit expression of the diffusion kernel, D(t), is required. The expression, Equation (9), of the diffusion kernel is chosen to reproduce the time‐dependence of the mean square displacement of the two‐state model, where an SV alternates between free diffusion (unbound state) and confined diffusion within a harmonic trap (bound state), which is relevant to the crowded environment in the presynaptic terminal.^[^ ^29^, ^61^, ^62^ ^]^ In this model, SVs freely diffuse with the diffusion coefficient, *D_s_*, in the unbound state, but repeated transitions between the bound and unbound states cause the overall diffusion coefficient, *D*_∞_, to be smaller than *D_s_*.

*Solution of Transport Equation*: The solution of Equation (8) in free space can be obtained by applying the Fourier‐Laplace transform to both sides of Equation (8) and arranging the result with respect to ${\hat{\tilde{P}}}_{0}\left(k,s|F\right)$ as

(25) $${\hat{\tilde{P}}}_{0}\left(k,s|F\right)=\frac{1}{s+\hat{D}\left(s\right)\left[k^{2}-ik\beta F\right]}$$

where ${\tilde{P}}_{0}\left(k,t|F\right)$ denotes the Fourier transform of the distribution, *P*_0_(*z*,*t*|*F*), of the SV displacement, *z*, at time *t* for a given *F*, i.e., ${\tilde{P}}_{0}\left(k,t|F\right)=\int_{-\infty }^{\infty }dze^{ikz}P_{0}\left(z,t|F\right)$. The corresponding *n*th‐order moment, Δ_*n*,0_(*t*|*F*), of the SV displacement can then be calculated as $\Delta_{n,0}\left(t|F\right)=L_{s\to t}^{-1}\left[\lim_{k\to 0}i^{-n}\partial_{k}^{n}{\hat{\tilde{P}}}_{0}\left(k,s|F\right)\right]$ with $L_{s\to t}^{-1}$ denoting the inverse Laplace transformation. For the first‐ and second‐order moments, their analytic expressions are given by

(26) $$\Delta_{1,0}\left(t|F\right)=\beta F\tau \left(t\right)$$

(27) $$\Delta_{2,0}\left(t|F\right)=2\tau \left(t\right)+2{\left(\beta F\right)}^{2}\int_{0}^{t}dt^{\prime }\dot{\tau }\left(t^{\prime }\right)\tau \left(t-t^{\prime }\right)$$

where *τ*(*t*) is a function of *t*, defined by *τ*(*t*) = *D*_∞_ *t* − (*D*_∞_ − *D_s_*)(1 − *e* ^−*λ* *t*^)/*λ* and $\dot{\tau }\left(t\right)$ denotes its time derivative. On the right‐hand side of Equation (27), the second term can be exactly calculated as

(28) $$\begin{array}{ccc} & & \frac{2{\left(\beta F\right)}^{2}\left(3D_{\infty }-D_{s}\right)}{\lambda }\\ & & \times \left[\left(1+\frac{D_{\infty }}{2D_{s}}\frac{3D_{\infty }-2D_{s}}{3D_{\infty }-D_{s}}\lambda t\right)D_{s}t-\left(1+\frac{D_{\infty }-D_{s}}{3D_{\infty }-D_{s}}\lambda t\right)\tau \left(t\right)\right] \end{array}$$

The asymptotic expressions of Equations (26) and (27) are given by Equations (13) and (14), respectively.

In the presence of the absorbing boundary located at *z* = 0, an analytic expression of the conditional probability density, *P*(*z*, *t*|*z*_0_,*F*), that an SV initially located at *z*_0_(< 0) is found at *z* at time *t* for a given *F* can also be obtained by solving Equation (8) under the condition that *P*(0, *t*|*z*_0_,*F*) = 0 in the Laplace domain:

(29) $$\hat{P}\left(z,s|z_{0},F\right)=\frac{e^{\beta F(z-z_{0})/2}}{\kappa (s|F)}\left[e^{-\left|z-z_{0}\right|\kappa \left(s|F\right)/2\hat{D}\left(s\right)}-e^{\left(z+z_{0}\right)\kappa \left(s|F\right)/2\hat{D}\left(s\right)}\right]$$

with *κ*(*s*|*F*) defined by $\kappa \left(s|F\right)=\sqrt{{\left[\beta F\hat{D}\left(s\right)\right]}^{2}+4s\hat{D}\left(s\right)}$. The corresponding *n*th‐order moment, Δ_*n*_(*t*|*z*_0_,*F*), of the SV displacement is then given in the Laplace domain by ${\hat{\Delta }}_{n}\left(s|z_{0},F\right)=\int_{-\infty }^{0}dz{\left(z-z_{0}\right)}^{n}\hat{P}\left(z,s|z_{0},F\right)$. For the survival probability that an SV does not reach the absorbing boundary as of *t*, and the first‐and second‐order moments of the SV displacement, their analytic expressions are given by,

(30) $${\hat{P}}_{S}\left(s|z_{0},F\right)={\hat{\Delta }}_{0}\left(s|z_{0},F\right)=\frac{1-e^{-\beta Fz_{0}/2}e^{\kappa \left(s|F\right)z_{0}/2\hat{D}\left(s\right)}}{s}$$

(31) $${\hat{\Delta }}_{1}\left(s|z_{0},F\right)=\frac{\beta F\hat{D}\left(s\right)}{s}{\hat{P}}_{S}\left(s|z_{0},F\right)+z_{0}\frac{1-s{\hat{P}}_{S}\left(s|z_{0},F\right)}{s}$$

(32) $$\begin{array}{c} {\hat{\Delta }}_{2}\left(s|z_{0},F\right)=\left(\frac{2\hat{D}\left(s\right)}{s^{2}}+\frac{2{\left[\beta F\hat{D}\left(s\right)\right]}^{2}}{s^{3}}\right){\hat{P}}_{S}\left(s|z_{0},F\right)\\ \;\;\;\;\;\;\;\;\;\;\;\;\;\;\;\;\;\;\;\;\;\;\;\;\;\;\;-z_{0}\left(z_{0}-\frac{2\beta F\hat{D}\left(s\right)}{s}\right)\frac{1-s{\hat{P}}_{S}\left(s|z_{0},F\right)}{s} \end{array}$$

Each model described in Figure 4A is characterized by the joint probability density, *p*_*α*_(*z*_0_,*F*), of the mean force (*F*) and initial position (*z*_0_); for Model *α* ∈ {I, II, III}, its expression is given by

(33) $$p_{I}\left(z_{0},F\right)=\delta \left(F\right)p_{a_{z_{0}},b_{z_{0}}}\left(z_{0}\right)$$

(34) $$p_{\mathrm{II}}\left(z_{0},F\right)=\delta \left(F-F_{c}\right)p_{a_{z_{0}},b_{z_{0}}}\left(z_{0}\right)$$

(35) $$p_{\mathrm{III}}\left(z_{0},F\right)=p_{a_{F},b_{F}\left(z_{0}\right)}\left(F|z_{0}\right)p_{a_{z_{0}},b_{z_{0}}}\left(z_{0}\right)\;\left(\mathrm{post}-\mathrm{stimulation}\mathrm{case}\right)$$

(36) $$p_{\mathrm{III}}\left(F\right)=p_{a_{F},b_{F}}\left(F\right)\;\left(\mathrm{pre}-\mathrm{stimulation}\mathrm{case}\right)$$

where $p_{a_{x},b_{x}}\left(x\right)$ denotes the marginal distribution of *x* ∈ {*z*_0_,*F*}, given by a gamma distribution, $p_{a_{x},b_{x}}\left(x\right)=|x{|}^{a_{x}-1}e^{-\left|x\right|/b_{x}}/\Gamma \left(a_{x}\right)b_{x}^{a_{x}}$. In Equation (34), *F_c_* is a constant mean force acting in the positive direction. In Equation (35), *p*(*F*|*z*_0_)$\left[=p_{a_{F},b_{F}\left(z_{0}\right)}\left(F\right)\right]$ denotes the gamma distribution of the mean force, conditioned on *z*_0_, whose expectation value $\bar{F}\left[=a_{F}b_{F}\left(z_{0}\right)\right]$ depends on *z*_0_ in a sigmoidal manner, i.e., $\bar{F}\left(z_{0}\right)=p_{<}\left(z_{0}\right)F_{<}+p_{>}\left(z_{0}\right)F_{>}$ with *p*_>_(*z*_0_) + *p*_<_(*z*_0_) = 1 and *p*_>_(*z*_0_)= (1 + exp [− 2*γ*(|*z*_0_| − *l_c_*)])^−1^. The value of $\bar{F}\left(z_{0}\right)$ undergoes a transition from *F*_<_ to *F*_>_(> *F*_<_ > 0) with a slope of (*F*_>_ − *F*_<_)*γ*/2 at |*z*_0_| = *l_c_* as the value of |*z*_0_| increases, passing through *l_c_*.

The distribution of initial distances (|*z*_0_|) for Type II SVs is best fit by a gamma distribution $p_{a_{z_{0}},b_{z_{0}}}\left(z_{0}\right)$ with $a_{z_{0}}=2.35$ and $b_{z_{0}}=8.92\times 10$ nm (Figure S8, Supporting Information). The theoretical results averaged over both *z*_0_ and *F* are calculated using the following equation:

(37) $$\Delta_{n}^{\left(\alpha \right)}\left(t\right)=\int_{-\infty }^{0}dz_{0}\int_{0}^{\infty }dF\Delta_{n}\left(t|z_{0},F\right)p_{\alpha }\left(z_{0},F\right)$$

in the post‐stimulation case. In the pre‐stimulation case without any boundary, the first two moments of the SV displacement for Model III are given by

(38) $$\Delta_{n,0}^{\left(\mathrm{III}\right)}\left(t\right)=\int_{0}^{\infty }dF\Delta_{n,0}\left(t|F\right)p_{\mathrm{III}}\left(F\right)\;\left(n=1,2\right)$$

with Δ_1,0_(*t*|*F*) and Δ_2,0_(*t*|*F*) given by Equations (26), and (27). The short‐ and long‐time asymptotic behaviors of the mean and variance of the SV displacement, given in Equations (13) and (14), are obtained from $\Delta_{1,0}^{\left(\mathrm{III}\right)}\left(t\right)$ and $\Delta_{2,0}^{\left(\mathrm{III}\right)}\left(t\right)-\Delta_{1,0}^{\left(\mathrm{III}\right)}{\left(t\right)}^{2}$.

Since the position of a QD within an SV was tracked in the experiment, it is necessary to account for the effect of intravesicle QD fluctuations (Text S5, Supporting Information). For Model *α* ∈ {I, II, III}, the mean SV displacement is given by $\Delta_{1}^{\left(\alpha \right)}\left(t\right)$ without modification, but the variance, $\sigma_{\alpha }^{2}\left(t\right)$, should be adjusted as

(39) $$\sigma_{\alpha }^{2}\left(t\right)=\Delta_{2}^{\left(\alpha \right)}\left(t\right)-\Delta_{1}^{\left(\alpha \right)}{\left(t\right)}^{2}/\Delta_{0}^{\left(\alpha \right)}\left(t\right)+2\left\langle \delta z_{QS}^{2}\right\rangle \Delta_{0}^{\left(\alpha \right)}\left(t\right)$$

with ${\left\langle \delta z_{QS}^{2}\right\rangle }^{1/2}$ denoting the magnitude of QD fluctuations centered at a given SV position. In the pre‐stimulation case without any boundary, $\Delta_{0}^{\left(\alpha \right)}\left(t\right)$ equals unity in Equation (39).

The initial distance dependence of the mean first passage time can provide an important information about the underlying SV transport dynamics. The mean first passage time, $\tau_{FP}^{\left(\alpha \right)}\left(z_{0}\right)$, dependent on the initial distance, is calculated as the whole time integration of the survival probability, $\Delta_{0}^{\left(\alpha \right)}\left(t|z_{0}\right)$, averaged over the conditional distribution, *p*_*α*_(*F*|*z*_0_)$\left[\equiv p_{\alpha }\left(z_{0},F\right)/p_{a_{z_{0}},b_{z_{0}}}\left(z_{0}\right)\right]$, i.e.,

(40) $$\begin{array}{c} \tau_{FP}^{\left(\alpha \right)}\left(z_{0}\right)=\int_{0}^{\infty }dt\Delta_{0}^{\left(\alpha \right)}\left(t|z_{0}\right)\\ \;\;\;\;\;\;\;\;\;\;\;\;\;\;\;\;=\int_{0}^{\infty }dt\left[\int_{0}^{\infty }dF\Delta_{0}\left(t|z_{0},F\right)p_{\alpha }\left(F|z_{0}\right)\right]\\ \;\;\;\;\;\;\;\;\;\;\;\;\;\;\;\;=\int_{0}^{\infty }dF\left[\int_{0}^{\infty }dt\Delta_{0}\left(t|z_{0},F\right)\right]p_{\alpha }\left(F|z_{0}\right)\\ \;\;\;\;\;\;\;\;\;\;\;\;\;\;\;\;=\int_{0}^{\infty }dF\tau_{FP}\left(z_{0},F\right)p_{\alpha }\left(F|z_{0}\right) \end{array}$$

where *τ*_*FP*_(*z*_0_,*F*) denotes the mean first passage time taken for an SV, initially located at *z*_0_, to first reach the absorbing boundary for a given *F*. An analytic expression of *τ*_*FP*_(*z*_0_,*F*), can be obtained by taking the small‐*s* limit of the survival probability, given in Equation (30):

(41) $$\tau_{FP}\left(z_{0},F\right)=\lim_{s\to 0}{\hat{\Delta }}_{0}\left(s|z_{0},F\right)=\frac{|z_{0}|}{\beta FD_{\infty }}$$

which shows that Equations (38) or (39) holds only for Models II and III; for Model I, the mean first passage time diverges. Using Equations ((34), (35)), and (39), Equation (38) can be calculated as

(42) $$\tau_{FP}^{\left(\mathrm{II}\right)}\left(z_{0}\right)=\frac{|z_{0}|}{\beta F_{c}D_{\infty }}$$

(43) $$\tau_{FP}^{\left(\mathrm{III}\right)}\left(z_{0}\right)=\frac{|z_{0}|}{\beta F^{*}\left(z_{0}\right)D_{\infty }}$$

where F*(*z*_0_) denotes the most probable value or mode of the conditional mean force distribution, *p*_III_(*F*|*z*_0_), for Model III, i.e., F*(*z*_0_) = (*a_F_* − 1)*b_F_* (*z*_0_).

For a given initial distance, the mean straightness, $S_{z}^{\left(\alpha \right)}\left(=|z_{0}|/{\left\langle l_{z}\right\rangle }_{z_{0}}^{\left(\alpha \right)}\right)$, is inversely proportional to the mean first passage time. ${\left\langle l_{z}\right\rangle }_{z_{0}}^{\left(\alpha \right)}$ denotes the mean pathlength along the *z*‐axis for a given *z*_0_, which can be estimated as the product of the longitudinal path‐averaged speed, *ε*_*z*_/Δ*t*, and the mean first passage time:

(44) $${\left\langle l_{z}\right\rangle }_{z_{0}}^{\left(\alpha \right)}≅\frac{\varepsilon_{z}}{\Delta t}\tau_{FP}^{\left(\alpha \right)}\left(z_{0}\right)=\frac{2D_{z}}{\varepsilon_{z}}\tau_{FP}^{\left(\alpha \right)}\left(z_{0}\right)$$

where *ε*_*z*_ and *D_z_* denote, respectively, the mean jump length during the experimental time resolution Δ*t* and the diffusion coefficient, $\varepsilon_{z}^{2}/2\Delta t$, along the *z‐*axis. Here, the mean jump length, *ε*_*z*_, is calculated as 〈*l_z_*/*t*〉Δ*t*, where *l_z_* and *t* represent, respectively, the pathlength and the first passage time for a given SV trajectory in the post‐stimulation case. Note that the value, 4.27 × 10^3^ nm^2^ s^−1^, of *D_z_* is close to the value, 4.45 × 10^3^ nm^2^ s^−1^, of the short‐time SV diffusion coefficient, *D_s_*, obtained from the best fit of Model III (Table S1 and Table 2, Supporting Information). Using Equation (44), the scaled mean straightness, $S_{z}^{\left(\alpha \right)}/\varepsilon_{z}$, is obtained as

(45) $$\frac{S_{z}^{\left(\alpha \right)}}{\varepsilon_{z}}=\frac{|z_{0}|}{\varepsilon_{z}{\left\langle l_{z}\right\rangle }_{z_{0}}^{\left(\alpha \right)}}=\frac{|z_{0}|}{2D_{z}\tau_{FP}^{\left(\alpha \right)}\left(z_{0}\right)}$$

For each model, the survival probability $\Delta_{0}^{\left(\alpha \right)}\left(t\right)$, the survival probability‐weighted mean and variance, $\Delta_{1}^{\left(\alpha \right)}\left(t\right)$ and $\sigma_{\alpha }^{2}\left(t\right)$, of the SV displacement, the initial distance dependent mean first passage time $\tau_{FP}^{\left(\alpha \right)}\left(z_{0}\right)$, and the scaled mean straightness $S_{z}^{\left(\alpha \right)}/\varepsilon_{z}$ were simultaneously optimized using Equations ((30), (31), (32), (33), (34), (35)), (37), (39) (43), and (45) in the post‐stimulation case (see Figure 4B–G; Text S6 for more details, Supporting Information); for Model I, only $\Delta_{0}^{\left(I\right)}\left(t\right)$, $\Delta_{1}^{\left(I\right)}\left(t\right)$, and $\sigma_{I}^{2}\left(t\right)$ were considered due to its divergent mean first passage time. The numerical Laplace inversions of Equations ((30), (31), (32)) were performed using the Stehfest method.^[^ ^63^ ^]^ In the pre‐stimulation case, only the mean and variance of the SV displacement were optimized (Figure 4H,I). Equations (26), (27), (38), and (39) with $\Delta_{0}^{\left(\mathrm{III}\right)}\left(t\right)=1$ were used for both the pre‐stimulation optimization of Model III with Equations (36) and the boundary‐free, post‐stimulation prediction of Model III with its optimized parameter values from Table 2 and the resulting marginal distribution of *F*, *p*_III_(*F*)|_post‐stimulation_ $\left[=\int_{-\infty }^{0}dz_{0}p_{\mathrm{III}}\left(z_{0},F\right)\right]$.

*Approximate time‐domain solution*: The Laplace‐domain analytical solution of Equation (8) with the absorbing boundary is provided by Equation 29, but its time‐domain expression remains unavailable. However, an approximate time‐domain expression interpolating the exact short‐ and long‐time asymptotic behaviors can be derived from the time‐local, deconvoluted transport equation, given by

(46) $$\begin{array}{c} \partial_{t}P\left(z,t|z_{0},F\right)=\int_{0}^{t}dt^{\prime }D\left(t^{\prime }\right)\frac{\partial }{\partial z}\left[\frac{\partial }{\partial z}-\beta F\right]P\left(z,t-t^{\prime }|z_{0},F\right)\\ \;\;\;\;\;\;\;\;\;\;\;\;\;\;\;\;\;\;\;\;\;\;\;\;\;\;\;\;\;\;\;\;≅\left(\int_{0}^{t}dt^{\prime }D\left(t^{\prime }\right)\right)\frac{\partial }{\partial z}\left[\frac{\partial }{\partial z}-\beta F\right]P\left(z,t|z_{0},F\right) \end{array}$$

In the presence of the absorbing boundary at *z* = 0, the solution, P*(*z*, *t*|*z*_0_,*F*), of the deconvoluted equation in Equation (46) can be obtained as follows: (Text S4, Supporting Information

(47) $$\begin{array}{c} P^{*}\left(z,t|z_{0},F\right)=\frac{1}{\sqrt{4\pi \tau (t)}}\left[e^{-{\left[z-z_{0}-\beta F\tau \left(t\right)\right]}^{2}/4\tau \left(t\right)}-e^{\beta Fz}e^{{\left[-z+z_{0}+\beta F\tau \left(t\right)\right]}^{2}/4\tau \left(t\right)}\right] \end{array}$$

where τ(*t*) is identical to that used in Equation (26). The corresponding analytic expressions for the survival probability, and the first‐ and second‐order moments of the SV displacement are given by Equation ((10), (11), (12)).

*Initial distance dependence of fusion time*: The non‐monotonic initial distance (*L*_SF_) dependence of the fusion time can be explained based on the *z*_0_‐dependent mean first passage time $\tau_{FP}^{\left(\mathrm{III}\right)}\left(z_{0}\right)$ for Model III, given in Equation (43). Using the linear relationship between the distance, *L*_SF_, from the stimulation site to the fusion site and the distance, |*z*_0_|, from the stimulation site to the tethering site in the longitudinal direction (Figure S4, Supporting Information), which is given by *L*_SF_ = *A*|*z*_0_| + *B* with *A* = 0.943 and *B* = 7.18 × 10^−2^ µm, $\tau_{FP}^{\left(\mathrm{III}\right)}\left(z_{0}\right)$ can be written as a function of *L*_SF_, i.e., $\tau_{FP}^{\left(\mathrm{III}\right)}\left(z_{0}\right)$ $=\tau_{FP}^{\left(\mathrm{III}\right)}\left(\right|z_{0}\left|\right)$ $=\tau_{FP}^{\left(\mathrm{III}\right)}\left(A^{-1}\left(L_{\mathrm{SF}}-B\right)\right)$. The theoretical result, $\tau_{F}^{\left(\mathrm{III}\right)}\left(L_{\mathrm{SF}}\right)$, for the *L*_SF_‐dependent fusion time, shown in Figure 4J, is then obtained by adding the offset, *C*, to $\tau_{FP}^{\left(\mathrm{III}\right)}\left(A^{-1}\left(L_{\mathrm{SF}}-B\right)\right)$ with the optimized parameter values for Model III, presented in Table 2:

(48) $$\tau_{F}^{\left(\mathrm{III}\right)}\left(L_{\mathrm{SF}}\right)=\tau_{FP}^{\left(\mathrm{III}\right)}\left(A^{-1}\left(L_{\mathrm{SF}}-B\right)\right)+C$$

where the value of *C* is estimated as the difference, 30.6 s, between the mean fusion time and the mean first passage time.

## Conflict of Interest

The authors declare no conflict of interest.

## Supporting information



Supporting Information

## Data Availability

Research data are not shared.
