# Supplementary material for: Initial Distance‐Dependent Mean Force Drives Synaptic Vesicle Motion Toward Fusion Sites in Stimulated Hippocampal Neurons
Source: Adv Sci (Weinh). 2025 Dec 19;13(12):e13823. doi: 10.1002/advs.202513823 (PMC12948204; doi:10.1002/advs.202513823)
Supplement: Supplementary file 1 — Supporting Information [file ADVS-13-e13823-s001.docx]

Supporting Information

**Initial Distance Dependent Mean Force Driving Synaptic Vesicle Motion Toward Fusion Sites in Stimulated Hippocampal Neurons**

Gyunam Park^1,2,3†^, Ji-Hyun Kim^1,2†^, Hunki Lee^4^, Chungwon Park^5^, Sidong Chen^5^, Luke Bates^6^, Jaeyoung Sung^1,2,3*^ and Hyokeun Park^5,7,8*^

Gyunam Park*^1,2,3^*, Ji-Hyun Kim*^1,2^*, Jaeyoung Sung*^1,2,3^*

Global Science Research Center for Systems Chemistry, Chung-Ang University, Seoul 06974, Korea

E-mail: jaeyoung@cau.ac.kr

Gyunam Park*^1,2,3^*, Ji-Hyun Kim*^1,2^*, Jaeyoung Sung*^1,2,3^*

Creative Research Initiative Center for Chemical Dynamics in Living Cells, Chung-Ang University, Seoul 06974, Korea

Gyunam Park*^1,2,3^*, Jaeyoung Sung*^1,2,3^*

Department of Chemistry, Chung-Ang University, Seoul 06974, Korea

Hunki Lee*^4^*

Max-Planck Institute for Molecular Biomedicine, Röntgenstraße 20, 48149 Münster, Germany

Chungwon Park*^5^*, Sidong Chen*^5^*, Hyokeun Park*^5,7,8^*

Division of Life Science, The Hong Kong University of Science and Technology, Clear Water Bay, Kowloon, Hong Kong.

E-mail: hkpark@ust.hk

Luke Bates*^6^*

Department of Computer Science and Hessian Center for AI, Technical University of Darmstadt, Hochschulstraße 10, 64289 Darmstadt, Germany.

Hyokeun Park*^5,7,8^*

Department of Physics, The Hong Kong University of Science and Technology, Clear Water Bay, Kowloon, Hong Kong SAR, China.

Hyokeun Park*^5,7,8^*

State Key Laboratory of Nervous System Disorders, The Hong Kong University of Science and Technology, Clear Water Bay, Kowloon, Hong Kong SAR, China.

*^†^* Gyunam Park and Ji-Hyun Kim contributed equally to this work

Supplementary Text S1

Measurement of synaptic vesicle tethering time and identification of fusion domains

Synaptic vesicles (SVs) typically exhibit confined motion near their fusion sites before they undergo fusion with the plasma membrane.^[1-2]^ The onset time of this confined motion, just prior to fusion, is referred to as the tethering time and the difference between the fusion time and tethering time corresponds to the tethering lifetime. Subsequently, a three-dimensional spatial domain spanned by an SV trajectory between the tethering and fusion events is termed the fusion domain (**Figure S1A**).

To determine the tethering time and the corresponding fusion domain, we analyzed trajectories of SVs near their fusion sites. The tethering time is determined as the time at which the distance of an SV from its fusion site begins to abruptly increase when we track the trajectory in reverse from the fusion event (Figure S1B, C). We also use a convex hull to refine the determination of the tethering time, which is the smallest polygon enclosing the trajectory points between a specified time frame and the final time frame of the fusion event. To construct these convex hulls, we use the convhulln function embedded in MATLAB. Since the convex hull includes only the outermost points, the radius of gyration calculated from these points effectively captures abrupt increases in the size of the spatial domain enclosed by the convex hull (Figure S1D). Finally, when a single SV trajectory is segmented into two parts before and after a given time frame, we define the trajectory overlap index as the number of trajectory points from the earlier segment that fall within the convex hull of the later segment (Figure S1E, F).^[3]^ The minimum value of this index, excluding the two endpoints, is also a useful criterion for determining the tethering time.

The identified fusion domains appear reasonable based on their sizes and the variance of the SV displacement within the domains. The fusion domain size, estimated using the radius of gyration for trajectory points constituting the convex hull has a mean value of 117 ± 3.76 nm (mean ± standard error of the mean) (**Figure S2A**). This value is consistent with the reported axial positions of the N-terminals of bassoon and piccolo relative to the active zone.^[4]^ In addition, the variance of the SV displacement during the tethering lifetime exhibits rapid saturation within 1 second, in contrast with the pre-tethering variance, which continues to increase with time (Figure S2B). This behavior indicates that SVs within the identified fusion domains undergo confined motion, suggesting that they are tethered to these domains. The magnitude of the effective one-dimensional fluctuation in the tethered SV’s position, estimated from the square root of the saturated variance divided by the spatial dimensionality, which is three, amounts to 55 nm. This value is comparable to the typical SV diameter of approximately 40 nm,^[5]^ implying that the saturated variance primarily originates from intravesicle fluctuations of quantum dots (QDs).

Supplementary Text S2

Relationship between mean straightness and mean force

In order to calculate the mean straightness based on a continuous-time random walk (CTRW) model,^[6-10]^ let us consider a one-dimensional lattice along the *α*-axis with a lattice spacing of $\varepsilon_{\alpha}$, where a random walker begins its motion at $m=0$ and an absorbing boundary is located at $m=m_{L}(>0)$ with *m* being the site index. The distance between the initial position ($m=0$) and the absorbing boundary ($m=m_{L}$) is then given by $L=m_{L}\varepsilon_{\alpha}$. In addition, the mean, $\langle l_{\alpha}\rangle_{L}$, of trajectory length, $l_{\alpha}$, can be written as $\langle l_{\alpha}\rangle_{L}=\langle N\rangle_{L}\varepsilon_{\alpha}$, where $\langle N\rangle_{L}$ denotes the mean number of jumps the random walker makes before it reaches the absorbing boundary. The mean straightness, $S_{\alpha}$, can then be calculated as $S_{\alpha}=L/\langle l_{\alpha}\rangle_{L}=m_{L}/\langle N\rangle_{L}$.

$\langle N_{\alpha}\rangle_{L}$ can be calculated for the CTRW model characterized by the waiting time distribution, $\psi_{\alpha}(t)$, for a random walker’s jump to one of two nearest neighboring sites along the *α*-axis. The mean, $\langle N(t)\rangle$, of the jump number, *N*, at time *t* is related to $\psi_{\alpha}(t)$ as $\langle\hat{N}(s)\rangle=s^{-1}\hat{\psi}_{\alpha}(s)/[1-\hat{\psi}_{\alpha}(s)]$ in the Laplace domain, where $\hat{f}(s)$ denotes the Laplace transform of $f(t)$ with respect to *t*, i.e., $\hat{f}(s)=\int_{0}^{\infty} dte^{-st}f(t)$. For a waiting time distribution, whose first-order moment, $\tau_{\alpha}$, is finite, $\langle N(t)\rangle$ becomes linear in time at times even longer than $\tau_{\alpha}$, explicitly, $\langle N(t)\rangle\cong t/\tau_{\alpha}$. With $\tau_{FP,\alpha}(L)$ denoting the mean time taken for a random walker to reach $m=m_{L}$ for the first time, $\langle N\rangle_{L}$ can be estimated as $\langle N\rangle_{L}\cong\tau_{FP,\alpha}(L)/\tau_{\alpha}$, which is valid when $\tau_{FP,\alpha}(L)$ is even larger than $\tau_{\alpha}$, or exact when $\psi_{\alpha}(t)$ is given by an exponential distribution, i.e., $\psi_{\alpha}(t)=\tau_{\alpha}^{-1}e^{-t/\tau_{\alpha}}$.

The mean first passage time, $\tau_{FP,\alpha}(L)$, can be calculated with the following procedure. First, let us consider the probability, $p_{\alpha}(m,t)$, that a random walker is located at the *m-*th site at time *t*, which is given by^[11-12]^

$p_{\alpha}(m,t)=\sum_{N=|m|}^{\infty} p_{\alpha}(m|N)P_{\alpha,N}(t)$,

where $p_{\alpha}(m|N)$ denotes the conditional probability that the random walker is located at the *m-*th site, given that it has undergone *N* jumps, explicitly, $p_{\alpha}(m|N)=\left(N!/m_{+}!m_{-}!\right)p_{\alpha,-}^{m_{-}}p_{\alpha,+}^{m_{+}}$ with $m_{\pm}=(N\pm m)/2$.^[13]^ $p_{\alpha,+(-)}$ indicates the probability that the random walker makes a jump in the positive (negative) direction along the *α*-axis, here, which is explicitly given by $p_{\alpha,\pm}=1/2\pm\delta_{\alpha}$ ($0\leq\delta_{\alpha}\leq1/2$) with $\delta_{\alpha}$ being a parameter yielding a bias toward the positive direction. In Equation S1, $P_{\alpha,N}(t)$. denotes the probability that the total number of jumps made by a random walker is *N* at time *t*, which is related to $\psi_{\alpha}(t)$ by $\hat{P}_{\alpha,N}(s)=s^{-1}[1-\hat{\psi}_{\alpha}(s)]\hat{\psi}_{\alpha}(s)^{N}$ in the Laplace domain. Performing the summation over *N* on the right-hand side of Equation S1 in the Laplace domain, the resulting equation is given by

$\hat{p}_{\alpha}(m,s)=\frac{1-\hat{\psi}_{\alpha}(s)}{s}\frac{\left[(1+\text{sgn}(m)2\delta_{\alpha})\hat{h}(s)\right]^{|m|}}{\sqrt{1-4p_{\alpha,+}p_{\alpha,-}\hat{\psi}_{\alpha}(s)^{2}}};\text{ }\hat{h}(s)=\frac{\hat{\psi}_{\alpha}(s)}{1+\sqrt{1-4p_{\alpha,+}p_{\alpha,-}\hat{\psi}_{\alpha}(s)^{2}}}$,

where sgn(*m*) denotes the sign of *m*. Second, let $p_{\alpha}(m,t|L)$ denote the probability that a random walker is located at the *m-*th site at time *t* under the absorbing boundary condition at $m=m_{L}$, i.e., $p_{\alpha}(m_{L},t|L)=0$. Using the conventional image method,^[14]^ an analytical expression of $\hat{p}_{\alpha}(m,s|L)$ can be obtained as

$\hat{p}_{\alpha}(m,s|L)=\hat{p}_{\alpha}(m,s)-(p{}_{\alpha,+}/p{}_{\alpha,-})^{m_{L}}\hat{p}_{\alpha}(m-2m_{L},s)$.

The Laplace transform, $\hat{P}_{S,\alpha}(s|L)$, of the survival probability, $P_{\alpha}(t|L)$, that a random walker has not reached the absorbing boundary by time *t* is then obtained by summing Equation over *m*. The resulting equation is given by

$$\begin{matrix} \hat{P}_{S,\alpha}(s|L)=\sum_{m=-\infty}^{m_{L}} \hat{p}_{\alpha}(m,s|L) \\ =\frac{1-\hat{\psi}_{\alpha}(s)}{s}\frac{\left[1-2^{m_{L}}p_{\alpha,+}^{m_{L}}\hat{h}(s)^{m_{L}}\right]\left[1-4p_{\alpha,+}p_{\alpha,-}\hat{h}(s)^{2}\right]}{\sqrt{1-4p_{\alpha,+}p_{\alpha,-}\hat{\psi}_{\alpha}(s)^{2}}\left[1-2\hat{h}(s)+4p_{\alpha,+}p_{\alpha,-}\hat{h}(s)^{2}\right]}. \end{matrix}$$

By taking the small-*s* limit of Equation S4, we can obtain the mean first passage time, $\tau_{FP,\alpha}(L)$, as $\tau_{FP,\alpha}(L)=\hat{P}_{S,\alpha}(0|L)=(m_{L}/2\delta_{\alpha})\tau_{\alpha}$. Finally, $\langle N\rangle_{L}$ can be estimated as $\tau_{FP,\alpha}(L)/\tau_{\alpha}=m_{L}/2\delta_{\alpha}$, from which the mean straightness, $S_{\alpha}$, is obtained as

$S_{\alpha}=m_{L}/\langle N\rangle_{L}=2\delta_{\alpha}$,

which shows the direct relationship between the mean straightness and the bias parameter, $\delta_{\alpha}$, defined by $2\delta_{\alpha}=p_{\alpha,+}-p_{\alpha,-}$. Equation S5 tells us that the mean straightness is determined solely by the degree of bias in the random walk, independent of the first passage kinetics. For the fully biased case with $\delta_{\alpha}=1/2$, the value of $S_{\alpha}$ is given by unity, while for the purely random or unbiased case with $\delta_{\alpha}=0$, i.e., $p_{\alpha,+}=p_{\alpha,-}=1/2$, the value of $S_{\alpha}$ is given by zero. In addition, the mean first passage time is linearly proportional to the initial distance, making the mean straightness independent of the initial distance. Thus, Equation S5 remains valid even when the initial distance follows a specific statistics. It is also known that the magnitude, $F_{\alpha}$, of the gradient of the potential of mean force is related to $p_{\alpha,\pm}$ by with ,^[15-16]^ where $k_{B}$ and *T* denote the Boltzmann constant and the absolute temperature, respectively. Using the latter relation and Equation S5, $S_{\alpha}$ can be expressed in terms of $F_{\alpha}$ as

$S_{\alpha}=\beta F_{\alpha}\varepsilon_{\alpha}/2$.

The mean straightness can also be calculated from the mean velocity, $v_{\alpha}$, defined by the long-time increasing rate of the mean displacement, $\varepsilon_{\alpha}\langle m_{\alpha}(t)\rangle$, in the absence of absorbing boundary. Using Equation S2, the Laplace-domain expression of the mean displacement in the absence of boundary can be obtained as

$$\begin{matrix} \varepsilon_{\alpha}\langle\hat{m}_{\alpha}(s)\rangle=\varepsilon_{\alpha}\sum_{m=-\infty}^{\infty} m\hat{p}_{\alpha}(m,s) \\ =\frac{1-\hat{\psi}_{\alpha}(s)}{s}\frac{4\delta_{\alpha}\varepsilon_{\alpha}\hat{h}(s)}{\sqrt{1-4p_{\alpha,+}p_{\alpha,-}\hat{\psi}_{\alpha}(s)^{2}}}\frac{1-4p_{\alpha,+}p_{\alpha,-}\hat{h}(s)^{2}}{\left[1-2\hat{h}(s)+4p_{\alpha,+}p_{\alpha,-}\hat{h}(s)^{2}\right]^{2}}, \end{matrix}$$

which reduces to $\varepsilon_{\alpha}\langle\hat{m}_{\alpha}(s)\rangle\cong2\delta_{\alpha}\varepsilon_{\alpha}/\tau_{\alpha}s^{2}$ in the small-*s* limit. In other words, the mean displacement linearly increases with time at long times:

$\varepsilon_{\alpha}\langle m_{\alpha}(t)\rangle\cong\frac{(2\delta_{\alpha})\varepsilon_{\alpha}}{\tau_{\alpha}}t$.

From Equation S8, $v_{\alpha}$ is obtained as $v_{\alpha}=(2\delta_{\alpha})\varepsilon_{\alpha}/\tau_{\alpha}$. With this result at hand, Equation S5 can be rewritten as

$S_{\alpha}=\frac{v_{\alpha}\tau_{\alpha}}{\varepsilon_{\alpha}}=\frac{v_{\alpha}\varepsilon_{\alpha}}{2D_{\alpha}}$,

where $D_{\alpha}$ denotes the diffusion coefficient along the *α*-axis, defined by $D_{\alpha}=\varepsilon_{\alpha}^{2}/2\tau_{\alpha}$.^[13]^

$S_{\alpha}$ is dependent on the experimental time resolution because the lattice spacing, $\varepsilon_{\alpha}$, corresponds to the mean travel distance during the experimental time resolution, which is 0.1 seconds in our experiment. However, the value of $S_{\alpha}/\varepsilon_{\alpha}$ is independent of the time resolution as shown in Equation S6 or S9, i.e., $S_{\alpha}/\varepsilon_{\alpha}=\beta F_{\alpha}/2$ or $v_{\alpha}/2D_{\alpha}$.

Supplementary Text S3

Relationship between diffusion kernel and reversible trapping model

In our SV transport equation, given by Equation 8, the diffusion kernel plays a central role in capturing the complex dynamics of SV motion. To determine its explicit time dependence, we consider a two-state model in which SVs undergo reversible trapping in the presynaptic terminal, which has been observed using various experimental methods.^[17-19]^

In the two-state model, an SV freely diffuses along the longitudinal *z*-axis with a diffusion coefficient, *D*, in the unbound state. In the bound state, the SV undergoes an Ornstein-Uhlenbeck (OU) process,^[20]^ which describes the thermal motion of a Brownian particle diffusing with the same diffusion coefficient, *D*, but confined within a harmonic potential, , where *z*′ represents the SV’s position at the moment it undergoes a transition from the unbound state to the bound state. Reversible transitions between the two states occur with rate constants, $k_{b}$ and $k_{u}$, representing the transition rates from the bound state to the unbound state and vice versa. In each state, the time evolution of the Green’s function, $G_{u(b)}(z,t)$, which represents the probability density of an SV being displaced by $z$ after time $t$ since the SV state transitions from the bound (unbound) state to the unbound (bound) state is governed by the following equation:

$\frac{\partial}{\partial t}G_{u}(z,t)=D\frac{\partial^{2}}{\partial z^{2}}G_{u}(z,t)-k_{u}G_{u}(z,t)$,

$\frac{\partial}{\partial t}G_{b}(z,t)=D\frac{\partial^{2}}{\partial z^{2}}G_{b}(z,t)+\rho\frac{\partial}{\partial z}zG_{b}(z,t)-k_{b}G_{b}(z,t)$, (S10B)

where *ρ* indicates the relaxation rate for the OU process, related to *D* and *a* by $\rho=Da/k_{B}T$ with $k_{B}$ and $T$ denoting the Boltzmann constant and temperature, respectively. Applying the Fourier-Laplace transform, $\int_{-\infty}^{\infty} dxe^{ikz}\int_{0}^{\infty} dte^{-st}(\cdots)$, to Equation S10A and rearranging the resulting equation, we obtain

$\hat{\tilde{G}}_{u}(k,s)=\frac{1}{s+k_{u}+Dk^{2}}$,

where $\tilde{g}(k)$ and $\hat{f}(s)$ denote, respectively, the Fourier transform of $g(z)$ with respect to *z* and the Laplace transform of $f(t)$ with respect to *t*, explicitly, $\tilde{g}(k)=\int_{-\infty}^{\infty} dxe^{ikz}g(z)$ and $\hat{f}(s)=\int_{0}^{\infty} dte^{-st}f(t)$. The solution to Equation S10B is simply given by the Green’s function for the OU process with the initial condition, $z_{0}$, multiplied by the probability, $e^{-k_{b}t}$, that an SV remains in the bound state without transitioning to the unbound state by time *t*, i.e.,

$G_{b}(z,t|z_{0})=\frac{e^{-k_{b}t}}{\sqrt{2\pi(D/\rho)(1-e^{-2\rho t})}}\exp\left[-\frac{(z-z_{0}e^{-\rho t})^{2}}{2(D/\rho)(1-e^{-2\rho t})}\right]$.

When $z_{0}=0$, meaning that *z* represents the SV displacement, the Fourier-Laplace transform of $G_{b}(z,t)\left[\equiv G_{b}(z,t|0)\right]$, given in Equation S12A, can be obtained as

$\hat{\tilde{G}}_{b}(k,s)=\frac{e^{-Dk^{2}/2\rho}}{2\rho}\frac{\gamma\left((s+k_{b})/2\rho,-Dk^{2}/2\rho\right)}{(-Dk^{2}/2\rho)^{(s+k_{b})/2\rho}}$ (S12B)

with $\gamma(a,z)$ being the lower incomplete gamma function, defined by $\gamma(a,z)=\int_{0}^{z} dt t^{a-1}e^{-t}$.

The Fourier-Laplace transform, $\hat{\tilde{p}}(k,s)$, of the displacement distribution, $p(z,t)$, can be constructed using $\hat{\tilde{G}}_{u}(k,s)$ and $\hat{\tilde{G}}_{b}(k,s)$ as follows:

$\hat{\tilde{p}}(k,s)=\mathbf{1}^{T}\cdot\hat{\tilde{G}}(k,s)\cdot\mathbf{p}_{0}$,

where **1** and $\mathbf{p}_{0}$ are the two-dimensional column vectors; the former’s elements are all unity and the latter’s elements are the initial stationary fractions of the unbound and bound states, i.e., $\mathbf{p}_{0}^{T}=(k_{b}/(k_{u}+k_{b}),k_{u}/(k_{u}+k_{b}))$. *T* denotes the transpose. In Equation S13, $\hat{\tilde{G}}(k,s)$ is the (2×2)-dimensional matrix, whose elements are the Fourier-Laplace transforms of the multi-time joint probability densities, constructed through multiple space-time convolutions of individual Green’s functions to account for all possible state transition histories. This matrix is defined by

$\hat{\tilde{G}}(k,s)=\sum_{n=0}^{\infty} \hat{\tilde{G}}^{(n)}(k,s)$,

where $\hat{\tilde{G}}^{(n)}(k,s)$ represents the component associated with *n*-time transitions. When *n* is not equal to zero, $\hat{\tilde{G}}^{(n)}(k,s)$, is given by

$\hat{\tilde{G}}^{(n)}(k,s)=\hat{\tilde{S}}(k,s)\cdot\hat{\tilde{T}}(k,s)^{n-1}\cdot\hat{\tilde{T}}^{(0)}(k,s)$,

where $\hat{\tilde{S}}(k,s)$ and $\hat{\tilde{T}}(k,s)$ are the (2×2)-dimensional matrices, defined by

$\hat{\tilde{S}}(k,s)=\left(\begin{matrix} \hat{\tilde{G}}_{u}(k,s) & 0 \\ 0 & \hat{\tilde{G}}_{b}(k,s) \end{matrix}\right)$,

$\hat{\tilde{T}}(k,s)=\left(\begin{matrix} 0 & k_{b}\hat{\tilde{G}}_{b}(k,s) \\ k_{u}\hat{\tilde{G}}_{u}(k,s) & 0 \end{matrix}\right)$, (S16B)

where $k_{b(u)}\hat{\tilde{G}}_{b(u)}(k,s)$ denotes the Fourier-Laplace transform of $k_{b(u)}G_{b(u)}(z,t)$, which is the probability density that an SV is displaced by *z* and it undergoes a transition to the unbound (bound) state at time *t* since the bound (unbound) state just began. In Equation S14 and S15, $\hat{\tilde{G}}^{(0)}(k,s)$ and $\hat{\tilde{T}}^{(0)}(k,s)$ are the initial counterparts of $\hat{\tilde{S}}(k,s)$ and $\hat{\tilde{T}}(k,s)$, defined by

$\hat{\tilde{G}}^{(0)}(k,s)=\left(\begin{matrix} \hat{\tilde{G}}_{u}^{(0)}(k,s) & 0 \\ 0 & \hat{\tilde{G}}_{b}^{(0)}(k,s) \end{matrix}\right)$,

$\hat{\tilde{T}}^{(0)}(k,s)=\left(\begin{matrix} 0 & k_{b}\hat{\tilde{G}}_{b}^{(0)}(k,s) \\ k_{u}\hat{\tilde{G}}_{u}^{(0)}(k,s) & 0 \end{matrix}\right)$. (S17B)

As an initial preparation, we can consider two cases; first, the bound (unbound) state begins precisely at time zero, and second, the observation starts at a random time after the system has reached its stationary state following multiple state transitions. The second case corresponds to the stationary initial condition, in which the bound (unbound) state began prior to time zero. In our model, the unbound-state Green’s function in the Fourier-Laplace domain is identical for both initial conditions, meaning that $\hat{\tilde{G}}_{u}^{(0)}(k,s)$ is the same as $\hat{\tilde{G}}_{u}(k,s)$, given by Equation S11. However, the bound-state Green’s function in the Fourier-Laplace domain depends on the initial condition; for the first initial condition, $\hat{\tilde{G}}_{b}^{(0)}(k,s)$ is the same as $\hat{\tilde{G}}_{b}(k,s)$, given by Equation S12B, while for the stationary initial condition, $\hat{\tilde{G}}_{b}^{(0)}(k,s)$ differs from $\hat{\tilde{G}}_{b}(k,s)$. An analytic expression for $\tilde{G}_{b}^{(0)}(k,t)$ under the stationary initial condition can be obtained as

,

where $G_{b}(z,t|z')$ is given by Equation S12A and $_{1}F_{1}(a,b,z)$ denotes the confluent hypergeometric function. On the right-hand side of the first equality in Equation S18, the time zero for $G_{b}(z',t'|0)$ marks the moment at which an SV transitions from the unbound state to the bound state, after which the SV fluctuates within the trap for *t*′ without undergoing any further transitions. Subsequently, *z*′ and *z* − *z*′ denote, respectively, the SV position at time *t*′, when observation begins, and the SV displacement over time *t* following the start of the observation. *k* is the Fourier variable associated with the displacement, *z* − *z*′. On the right-hand side of the second equality in Equation S18, the integration over *t*′ represents the average of the Fourier transform over the distribution, $k_{b}e^{-k_{b}t'}$, of the probability that the SV remains in the trap without transitioning for *t*′.

Finally, with Equation S14 to S17 at hand, equation S13 can be expressed as

$$\begin{matrix} \hat{\tilde{p}}(k,s)=\mathbf{1}^{T}\cdot\left[\hat{\tilde{G}}^{(0)}(k,s)+\hat{\tilde{S}}(k,s)\cdot\sum_{n=1}^{\infty} \hat{\tilde{T}}(k,s)^{n-1}\cdot\hat{\tilde{T}}^{(0)}(k,s)\right]\cdot\mathbf{p}_{0} \\ =\mathbf{1}^{T}\cdot\left[\hat{\tilde{G}}^{(0)}(k,s)+\hat{\tilde{S}}(k,s)\cdot[I-\hat{\tilde{T}}(k,s)]^{-1}\cdot\hat{\tilde{T}}^{(0)}(k,s)\right]\cdot\mathbf{p}_{0} \\ =\frac{(1+k_{u}\hat{\tilde{G}}_{b}(k,s))k_{b}\hat{\tilde{G}}_{u}^{(0)}(k,s)+(1+k_{b}\hat{\tilde{G}}_{u}(k,s))k_{u}\hat{\tilde{G}}_{b}^{(0)}(k,s)}{(k_{u}+k_{b})(1-k_{u}k_{b}\hat{\tilde{G}}_{u}(k,s)\hat{\tilde{G}}_{b}(k,s))}, \end{matrix}$$

where the second equality follows from the matrix geometric series. The mean square displacement (MSD), $\Delta_{2}^{\text{two-state}}(t)\left[\equiv\int_{-\infty}^{\infty} dzz^{2}p(z,t)\right]$, for the two-state model is related to $\hat{\tilde{p}}(k,s)$ by $\Delta_{2}^{\text{two-state}}(t)=-\lim_{k\to0} \partial_{k}^{2}\tilde{p}(k,t)$. For the stationary initial condition, where $\hat{\tilde{G}}_{b}^{(0)}(k,s)$ is given not by Equation S12B but by the Laplace transform of Equation S18, taking the second-order derivative of Equation S19 with respect to *k* and performing the inverse Laplace transform of the resulting equation in the small-*k* limit, we obtain

$\Delta_{2}^{\text{two-state}}(t)=2D\left[\frac{k_{b}(k_{b}+k_{u}+2\rho)}{(k_{b}+k_{u})(k_{b}+2\rho)}t+\frac{1}{k_{b}+\rho}\left(1-\frac{k_{b}(k_{b}+k_{u}+2\rho)}{(k_{b}+k_{u})(k_{b}+2\rho)}\right)(1-e^{-(k_{b}+\rho)t})\right]$.

The expression, Equation 9, of the diffusion kernel is chosen to reproduce the time-dependence of the MSD, Equation S20, of the two-state model in the unbiased case, i.e., *F* = 0. The corresponding MSD, derived from our SV transport equation, Equation 8, with the diffusion kernel, Equation 9, is given by Equation 24B with *F* = 0:

$\Delta_{2,0}(t|F=0)=2D_{s}\left[\frac{D_{\infty}}{D_{s}}t+\frac{1}{\lambda}\left(1-\frac{D_{\infty}}{D_{s}}\right)(1-e^{-\lambda t})\right]$.

Note that Equation S20 and S21 share exactly the same time-dependence. Comparing these two equations, the parameters in the two-state model can be mapped into those in the diffusion kernel, explicitly,

$D_{s}=D$,

$D_{\infty}=D\frac{k_{b}(k_{b}+k_{u}+2\rho)}{(k_{b}+k_{u})(k_{b}+2\rho)}$, (S22B)

$\lambda=k_{b}+\rho$. (S22C)

Supplementary Text S4

Solutions to our SV transport equation

To describe the motion of synaptic vesicles (SVs), we establish a generalized transport equation, given by Equation 8. For SVs moving in free space, representing their motion prior to electrical stimulation, the corresponding solution to Equation 8 is denoted as $\hat{P}_{0}(z,s|F)$, which is the Laplace transform of the conditional probability density, $P_{0}(z,t|z_{0}=0,F)$, that an SV is found at position *z* at time *t* under a potential field, −*Fz*, given that it was initially located at $z_{0}=0$. With this initial condition, *z* corresponds to the SV’s displacement. Applying the Fourier transformation, $\int_{-\infty}^{\infty} dze^{ikz}(\cdots)$, to both sides of Equation 8 and rearranging the resulting equation with respect to $\hat{\tilde{P}}_{0}(k,s|F)$, we obtain

.

The *n-*th-order moment, $\hat{\Delta}_{n,0}(s|F)$, of the free-space SV displacement in the Laplace domain can then be obtained by using the formula, $\hat{\Delta}_{n,0}(s|F)=\int_{-\infty}^{\infty} dzz^{n}\hat{P}_{0}(z,s|F)$ $=\lim_{k\to0} i^{-n}\partial_{k}^{n}\hat{\tilde{P}}_{0}(k,s|F)$. The explicit expressions of $\hat{\Delta}_{1,0}(s|F)$ and $\hat{\Delta}_{2,0}(s|F)$ are given by

,

, (S24B)

Applying the inverse Laplace transform to Equation S24A and S24B results in

$\Delta_{1,0}(t|F)=\beta FD_{\infty}t+\beta F(D_{s}-D_{\infty})g(t)$,

$\begin{matrix} \Delta_{2,0}(t|F)=2D_{\infty}t+2(D_{s}-D_{\infty})g(t)+(\beta FD_{\infty}t)^{2} \\ +2(\beta F)^{2}(D_{s}-D_{\infty})\left[(D_{s}-D_{\infty})g(t)t+(D_{s}-3D_{\infty})(g(t)-t)/\lambda\right], \end{matrix}$ (S25B)

where $g(t)$ denotes a function of time, defined by $g(t)=(1-e^{-\lambda t})/\lambda$. When the mean force *F* follows a specific distribution, we need to average Equation S25A and S25B over the distribution. The resulting equations are given by

$$\Delta_{1,0}(t)=\beta\langle F\rangle D_{\infty}t+\beta\langle F\rangle(D_{s}-D_{\infty})g(t),$$

$\begin{matrix} \Delta_{2,0}(t)-\Delta_{1,0}^{2}(t)=2D_{\infty}t+2(D_{s}-D_{\infty})g(t)+\beta^{2}\langle\delta F^{2}\rangle(D_{\infty}t)^{2} \\ -\beta^{2}\langle F\rangle^{2}(D_{s}-D_{\infty})\left[2D_{\infty}t+(D_{s}-D_{\infty})g(t)\right]g(t) \\ +2\beta^{2}\langle F^{2}\rangle(D_{s}-D_{\infty})\left[(D_{s}-D_{\infty})g(t)t+(D_{s}-3D_{\infty})(g(t)-t)/\lambda\right]. \end{matrix}$ (S26B)

Note that the quadratic terms in *t* in Equation 14 and Equation S26B originate from the heterogeneity in the mean force; these terms are proportional to the variance, $\langle\delta F^{2}\rangle$, in *F*. Noting that $g(t)$ behaves as at short times and approaches $1/\lambda$ in the long-time limit, Equation 13 and 14 can be derived from Equation S26A and S26B.

In the presence of the absorbing boundary at *z* = 0, the corresponding solution to Equation 8 is denoted as $\hat{P}(z,s|z_{0},F)$, which is the Laplace transform of the conditional probability density, $P(z,t|z_{0},F)$, that an SV, initially located at $z_{0}(<0)$, is found at position *z* at time *t* under a potential field, −*Fz*, and subject to the absorbing boundary condition, $P(z=0,t|z_{0},F)=0$. An analytic expression of $\hat{P}(z,s|z_{0},F)$ can be obtained using the image method:^[14]^

with . The corresponding *n-*th-order moment of the SV displacement in the Laplace domain is calculated as $\hat{\Delta}_{n}(s|z_{0},F)=$ $\int_{-\infty}^{0} dz(z-z_{0})^{n}\hat{P}(z,s|z_{0},F)$. Analytic expressions of the survival probability, the first- and second-order moments in the Laplace domain are then given by

,

$\hat{\Delta}_{1}(s|z_{0},F)=\hat{\Delta}_{1,0}(s|F)s\hat{P}_{S}(s|z_{0},F)+z_{0}\frac{1-s\hat{P}_{S}(s|z_{0},F)}{s}$, (S28B)

$\hat{\Delta}_{2}(s|z_{0},F)=\hat{\Delta}_{2,0}(s|F)s\hat{P}_{S}(s|z_{0},F) -z_{0}\left(z_{0}-2s\hat{\Delta}_{1,0}(s|F)\right)\frac{1-s\hat{P}_{S}(s|z_{0},F)}{s}$, (S28C)

where $\hat{\Delta}_{1,0}(s|F)$ and $\hat{\Delta}_{2,0}(s|F)$ are given by Equations S24A and S24B. In the absence of the absorbing boundary, $\hat{P}_{S}(s|z_{0},F)$ becomes 1/*s*, causing the second terms on the right-hand sides of Equations S28B and S28C to vanish. Consequently, $\hat{\Delta}_{1}(s|z_{0},F)$ and $\hat{\Delta}_{2}(s|z_{0},F)$ reduce to their free-space counterparts. On the other hand, when the absorbing boundary is present, $P_{S}(t|z_{0},F)$ significantly modulates the time profile of $\Delta_{1(2)}(t|z_{0},F)$. Specifically, $\Delta_{1(2)}(t|z_{0},F)$ approaches zero in the long-time limit if the mean force magnitude is finite, in contrast with the free-space case where $\Delta_{1(2),0}(t|z_{0},F)$ continues to increase with time.

In an unbiased case, i.e., *F* = 0, $\hat{\Delta}_{1,0}(s|F)$ vanishes, as evident from Equation S24A, and hence $\hat{\Delta}_{1}(s|z_{0},F)$ simplifies to $z_{0}[1/s-\hat{P}_{S}(s|z_{0},F)]$. The corresponding time-domain expression is given by $\Delta_{1}(t|z_{0},F)=z_{0}[1-P_{S}(t|z_{0},F)]$, which is negative-definite because $z_{0}$ itself is negative, and $1-P_{S}(t|z_{0},F)$ is positive-definite except at time zero. Therefore, the observed positive mean displacement of surviving SVs, obtained from SV trajectories, strongly indicates the presence of an effective longitudinal force driving SVs toward their fusion sites.

The time-domain expressions of Equations S28A-C remain unavailable, but their approximate time-domain expressions interpolating the exact short- and long-time asymptotic behaviors can be derived from the time-local, deconvoluted transport equation, whose solution is denoted as $P^{*}(z,t|z_{0},F)$. The time-local transport equation is given by Equation 46, or

,

where corresponds to the time-dependent diffusion coefficient, $D(t)$. Using Equation 9, the explicit expression of $D(t)$ is obtained as

$D(t)=D_{\infty}+(D_{s}-D_{\infty})e^{-\lambda t}$,

which changes over time from the short-time limiting value, $D_{s}$, to the long-time limiting value,$D_{\infty}$. Introducing a new time variable, $\tau(t)=\int_{0}^{t} dt'D(t')/D_{\infty}$, and noting that $\partial P^{*}/\partial t$ $=(\partial P^{*}/\partial\tau)(\partial\tau/\partial t)$ $=(\partial P^{*}/\partial\tau)(D(t)/D_{\infty})$, Equation S29 can be rewritten as

$\frac{\partial}{\partial\tau}P^{*}(z,t|z_{0},F)=D_{\infty}\frac{\partial}{\partial z}\left(\frac{\partial}{\partial z}-\beta F\right)P^{*}(z,t|z_{0},F)$.

The solution to Equation S31 under the absorbing boundary condition at $z=0$ can be directly obtained from Equation 23 but with the Laplace-transformed diffusion kernel, , replaced with the long-time diffusion constant, $D_{\infty}$, which is explicitly given by

,

Note here that the Laplace variable, *s*, in Equation S32 corresponds to the time variable, *τ*. The inverse Laplace transformation of Equation S32 can be analytically performed as follows:^[14]^

$$P^{*}(z,t|z_{0},F)=\frac{1}{\sqrt{4\pi D_{\infty}\tau(t)}}\left[e^{-[z-z_{0}-\beta FD_{\infty}\tau(t)]^{2}/4D_{\infty}\tau(t)}-e^{\beta Fz}e^{-[z+z_{0}+\beta FD_{\infty}\tau(t)]^{2}/4D_{\infty}\tau(t)}\right]$$

with $\tau(t)=t+(D_{s}/D_{\infty}-1)(1-e^{-\lambda t})/\lambda$. The corresponding *n-*th-order moment of the SV displacement, defined by $\Delta_{n}^{*}(t|z_{0},F)=$ $\int_{-\infty}^{0} dz(z-z_{0})^{n}P^{*}(z,t|z_{0},F)$, is calculated for $n=$0, 1, and 2:

$P_{S}^{*}(t|z_{0},F)\equiv\Delta_{0}^{*}(t|z_{0},F)=C_{+}(t|z_{0},F)-e^{-\beta Fz_{0}}C_{-}(t|z_{0},F)$,

$\Delta_{1}^{*}(t|z_{0},F)=\left[\beta FD_{\infty}\tau(t)\right]P_{S}^{*}(t|z_{0},F)+2z_{0}e^{-\beta Fz_{0}}C_{-}(t|z_{0},F)$, (S34B)

$\begin{matrix} \Delta_{2}^{*}(t|z_{0},F)=\left[2D_{\infty}\tau(t)+[\beta FD_{\infty}\tau(t)]^{2}\right]P_{S}^{*}(t|z_{0},F) \\ -4z_{0}\left[C_{-}(t|F,z_{0})[z_{0}-\beta FD_{\infty}\tau(t)]+\sqrt{D_{\infty}\tau(t)/4\pi}e^{-[z_{0}+\beta FD_{\infty}\tau(t)]^{2}/4D_{\infty}\tau(t)}\right], \end{matrix}$ (S34C)

where $C_{\pm}(t|z_{0},F)$ is defined by . Here, erf(z) denotes the error function defined by .

Supplementary Text S5

Effects of intravesicle QD fluctuations on SV displacement moments

Because the position of a QD within an SV was tracked in the experiment, it is necessary to determine how the moments of the SV displacement relate to the moments of the QD displacement. Let $\mathbf{r}_{QD}$ denote the vector indicating the position of a QD within an SV located at $\mathbf{r}_{SV}$. The QD position vector, $\mathbf{r}_{QD}$, can then be expressed as

$\mathbf{r}_{QD}=\mathbf{r}_{SV}+(\mathbf{r}_{QD}-\mathbf{r}_{SV})=\mathbf{r}_{SV}+\mathbf{r}_{QS}$,

where .$\mathbf{r}_{QS}$. denotes the position of the QD relative to $\mathbf{r}_{SV}$, i.e., $\mathbf{r}_{QS}=\mathbf{r}_{QD}-\mathbf{r}_{SV}$. $\mathbf{r}_{QS}$ exhibits confined motion centered at $\mathbf{r}_{SV}$ without directional preference. This motion can thus be modeled by treating its three components, $\alpha_{QS}\in\{x_{QS},y_{QS},z_{QS}\}$, as independent and identically distributed random variables, each following a stationary, Ornstein-Uhlenbeck (OU) process,^[20]^ $\partial_{t}\alpha_{QS}=-\lambda\alpha_{QS}+\eta_{\alpha_{QS}}(t)$, where *λ* and $\eta_{\alpha_{QS}}(t)$ denote, respectively, the relaxation rate and white noise term satisfying $\langle\eta_{\alpha_{QS}}(t)\eta_{\beta_{QS}}(t')\rangle=2D\delta_{\alpha_{QS}\beta_{QS}}\delta(t-t')$ $\left[\alpha_{QS},\beta_{QS}\in\{x_{QS},y_{QS},z_{QS}\}\right]$. The corresponding mean and time correlation function are given by $\langle\alpha_{QS}(t)\rangle=0$and $\langle\alpha_{QS}(t)\alpha_{QS}(t')\rangle=\langle\alpha_{QS}^{2}\rangle e^{-\lambda|t-t'|}$ with $\langle\alpha_{QS}^{2}\rangle$ being the stationary variance.

Based on Equation S35, the QD displacement, $\Delta\mathbf{r}_{QD}(t)[=\mathbf{r}_{QD}(t)-\mathbf{r}_{QD}(0)]$, can be decomposed into the two components, $\Delta\mathbf{r}_{SV}(t)[=\mathbf{r}_{SV}(t)-\mathbf{r}_{SV}(0)]$ and $\Delta\mathbf{r}_{QS}(t)[=\mathbf{r}_{QS}(t)-\mathbf{r}_{QS}(0)]$, i.e., $\Delta\mathbf{r}_{QD}(t)=\Delta\mathbf{r}_{SV}(t)+\Delta\mathbf{r}_{QS}(t)$. The first-order moment of the QD displacement is then given by

$\langle\Delta\mathbf{r}_{QD}(t)\rangle=\langle\Delta\mathbf{r}_{SV}(t)\rangle+\langle\Delta\mathbf{r}_{QS}(t)\rangle=\langle\Delta\mathbf{r}_{SV}(t)\rangle$,

where we assume that $\mathbf{r}_{QS}$ exhibits symmetric fluctuations centered at $\mathbf{r}_{SV}$, resulting in$\langle\mathbf{r}_{QS}(t)\rangle=\mathbf{0}$, or $\langle\alpha_{QS}(t)\rangle=0$, at all times, which indicates that $\langle\Delta\mathbf{r}_{QS}(t)\rangle=\langle\mathbf{r}_{QS}(t)\rangle-\langle\mathbf{r}_{QS}(0)\rangle=\mathbf{0}$. On the other hand, the second-order moment of the QD displacement is given by

$$\begin{matrix} \langle\Delta\mathbf{r}_{QD}(t)^{2}\rangle=\langle\Delta\mathbf{r}_{SV}(t)^{2}\rangle+2\langle\Delta\mathbf{r}_{SV}(t)\cdot\Delta\mathbf{r}_{QS}(t)\rangle+\langle\Delta\mathbf{r}_{QS}(t)^{2}\rangle\\ =\langle\Delta\mathbf{r}_{SV}(t)^{2}\rangle+\langle\Delta\mathbf{r}_{QS}(t)^{2}\rangle, \end{matrix}$$

where we assume that the intravesicle QD fluctuations are independent of the SV transport dynamics. Consequently, $\Delta\mathbf{r}_{QS}$ evolves over time in a manner independent of $\Delta\mathbf{r}_{SV}(t)$, i.e., $\langle\Delta\mathbf{r}_{SV}(t)\cdot\Delta\mathbf{r}_{QS}(t)\rangle=\langle\Delta\mathbf{r}_{SV}(t)\rangle\cdot\langle\Delta\mathbf{r}_{QS}(t)\rangle$, which vanishes because $\langle\Delta\mathbf{r}_{QS}(t)\rangle=\mathbf{0}$. In addition, we assume that the intravesicle QD fluctuations occur so rapidly that $\langle\Delta\mathbf{r}_{QS}(t)^{2}\rangle$ can be treated as a time-independent constant within the temporal resolution, 0.1 seconds, of our experiments. Based on the OU model described above, $\langle\Delta\mathbf{r}_{QS}(t)^{2}\rangle$ can be calculated as

$$\begin{matrix} \langle\Delta\mathbf{r}_{QS}(t)^{2}\rangle=\sum_{\alpha_{QS}\in\{x_{QS},y_{QS},z_{QS}\}} \langle(\alpha_{QS}(t)-\alpha_{QS}(0))^{2}\rangle\\ =\sum_{\alpha_{QS}\in\{x_{QS},y_{QS},z_{QS}\}} \langle\alpha_{QS}(t)^{2}\rangle-2\langle\alpha_{QS}(t)\alpha_{QS}(0)\rangle+\langle\alpha_{QS}(0)^{2}\rangle\\ =\sum_{\alpha_{QS}\in\{x_{QS},y_{QS},z_{QS}\}} 2\langle\alpha_{QS}^{2}\rangle-2\langle\alpha_{QS}(t)\alpha_{QS}(0)\rangle\\ =\sum_{\alpha_{QS}\in\{x_{QS},y_{QS},z_{QS}\}} 2\langle\alpha_{QS}^{2}\rangle(1-e^{-\lambda t}) \\ =2\langle\mathbf{r}_{QS}^{2}\rangle(1-e^{-\lambda t}) \\ \cong2\langle\delta\mathbf{r}_{QS}^{2}\rangle, \end{matrix}$$

which is valid when the relaxation time $\lambda^{-1}$ is much shorter than 0.1 seconds. In Equation S38, we have used the fact that $\sum_{\alpha_{QS}} \langle\alpha_{QS}^{2}\rangle=\langle\mathbf{r}_{QS}^{2}\rangle$ $=\langle(\mathbf{r}_{QS}-\langle\mathbf{r}_{QS}\rangle)^{2}\rangle=\langle\delta\mathbf{r}_{QS}^{2}\rangle$. With Equation S36, S37, and S38 at hand, the mean and variance of the QD displacement can be expressed as

$\langle\Delta\mathbf{r}_{QD}(t)\rangle=\langle\Delta\mathbf{r}_{SV}(t)\rangle$,

$\langle\Delta\mathbf{r}_{QD}(t)^{2}\rangle-\langle\Delta\mathbf{r}_{QD}(t)\rangle^{2}=\langle\Delta\mathbf{r}_{SV}(t)^{2}\rangle-\langle\Delta\mathbf{r}_{SV}(t)\rangle^{2}+2\langle\delta\mathbf{r}_{QS}^{2}\rangle$ (S39B)

with their longitudinal *z*-components given by

$\langle\Delta z_{QD}(t)\rangle=\langle\Delta z_{SV}(t)\rangle=\Delta_{1}(t)/P_{S}(t)$,

$\begin{matrix} \langle\Delta z_{QD}(t)^{2}\rangle-\langle\Delta z_{QD}(t)\rangle^{2}=\langle\Delta z_{SV}(t)^{2}\rangle-\langle\Delta z_{SV}(t)\rangle^{2}+2\langle\delta z_{QS}^{2}\rangle\\ =\Delta_{2}(t)/P_{S}(t)-(\Delta_{1}(t)/P_{S}(t))^{2}+2\langle\delta z_{QS}^{2}\rangle, \end{matrix}$ (S40B)

where $P_{S}(t)$ represents the survival probability that an SV has not reached an absorbing boundary by time *t*, and $\Delta_{1}(t)$ and $\Delta_{2}(t)$ denote the first- and second-order displacement moments of SVs that remain within the boundary at time *t*. In Equation S40, the brackets denote averages over the trajectories of SVs or QDs, with the number of trajectories decreasing monotonically over time due to the presence of the absorbing boundary. $\langle\Delta z_{SV}(t)\rangle$ and $\langle\Delta z_{SV}(t)^{2}\rangle$ can thus be expressed as $\Delta_{1}(t)/P_{S}(t)$ and $\Delta_{2}(t)/P_{S}(t)$, where $P_{S}(t)$ functions as the time-dependent normalization constant. Finally, the unnormalized, survival probability-weighted mean and variance of the QD displacement are obtained by multiplying the survival probability with Equations S40A and S40B:

$P_{S}(t)\langle\Delta z_{QD}(t)\rangle=\Delta_{1}(t)$,

$P_{S}(t)\left[\langle\Delta z_{QD}(t)^{2}\rangle-\langle\Delta z_{QD}(t)\rangle^{2}\right]=\Delta_{2}(t)-\Delta_{1}(t)^{2}/P_{S}(t)+2\langle\delta z_{QS}^{2}\rangle P_{S}(t),$ (S41B)

where the optimized value of $\langle\delta z_{QS}^{2}\rangle^{1/2}$ for each model is presented in Table 2 in the main text.

Supplementary Text S6

Quantitative analysis using SV transport models

We simultaneously optimized parameters for each model to fit the corresponding experimental data using the *fmincon* function embedded in MATLAB. For Models II and III, the optimization process includes a nonlinear constraint to ensure agreement with the experimental value, 0.236, for the Pearson correlation coefficient, $r_{t,z_{0}}$, between the first passage time and the initial distance (Figure 4D); $r_{t,z_{0}}$ is calculated within the observation domain, defined as the region where the first passage time is less than 120 seconds and the initial distance is less than 1.2 μm.


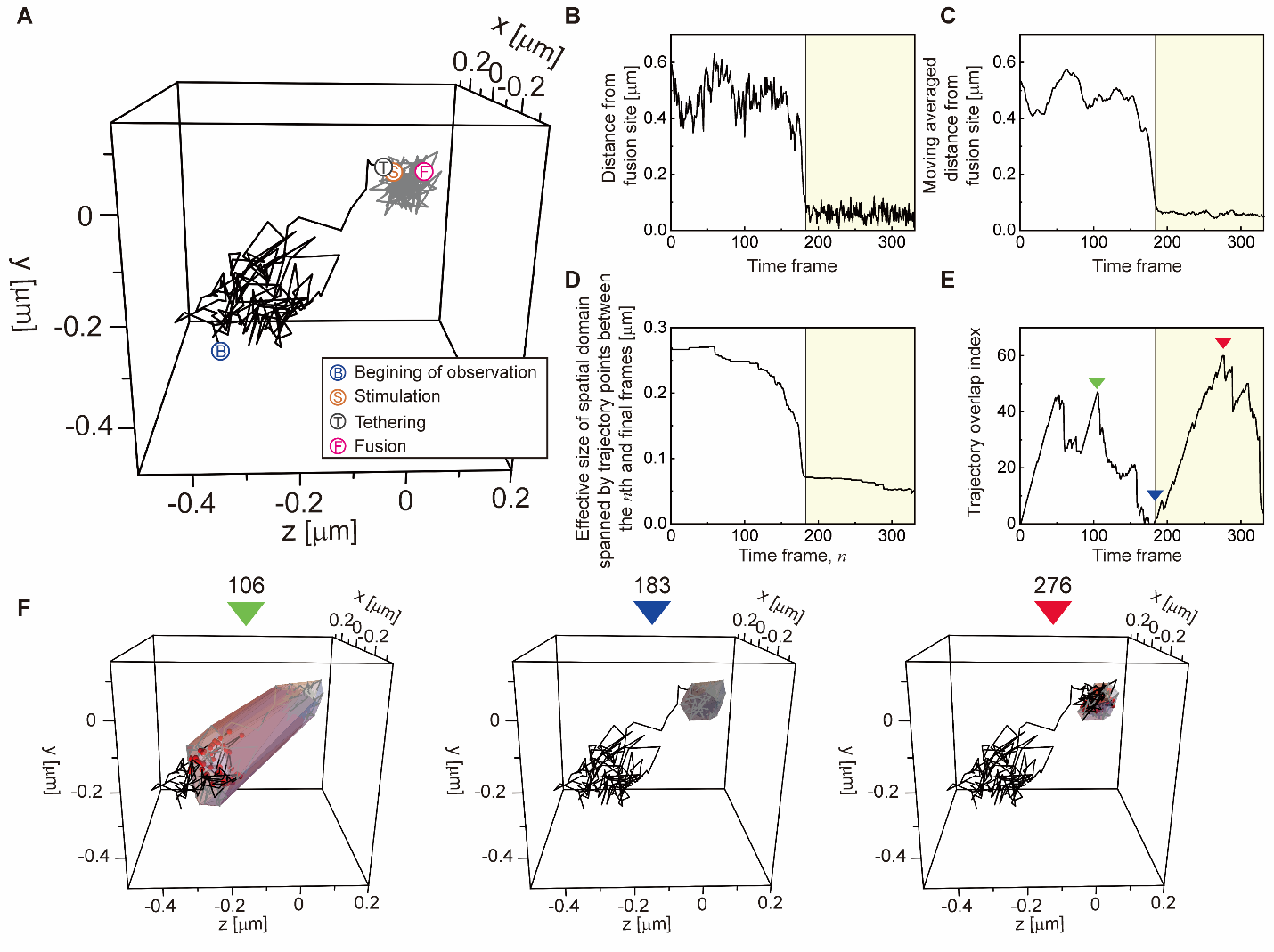


Figure S1. Measurement of synaptic vesicle tethering time and identification of fusion domains. A) A representative trajectory of a synaptic vesicle (SV) in the presynaptic terminal, showing a distinct transition from unconfined to confined motion, after the tethering event. The black and gray lines represent the pre-tethering and post-tethering segments of the SV trajectory, respectively. B) Distance from the fusion site to the SV position at each time frame. C) Smoothened time profile of the SV distance shown in B) using a one-second moving average. D) Effective size of the spatial domain spanned by trajectory points between the *n-*th and final time frames. The effective domain size is estimated as the radius of gyration calculated from the outermost points constituting the convex hull, which encloses all points between the *n-*th and final frames. E) Trajectory overlap index at each time frame, counting the number of points between the first and (*n*−1)-th frames that fall within the convex hull enclosing all points between the *n-*th and final frames. In B-E), the time frame at the boundary between the unshaded and yellow-shaded areas corresponds to the tethering time for the SV trajectory shown in A). The spatial domain spanned by the gray segments corresponds to the fusion domain. F) Convex hulls constructed at three different time frames. The red symbols indicate trajectory points between the first and (*n*−1)-th frames, which fall within the convex hull enclosing all points between the *n*th and final frames: *n* = 106, 183, and 276, which are marked by the green, blue, and red downward triangles in F).


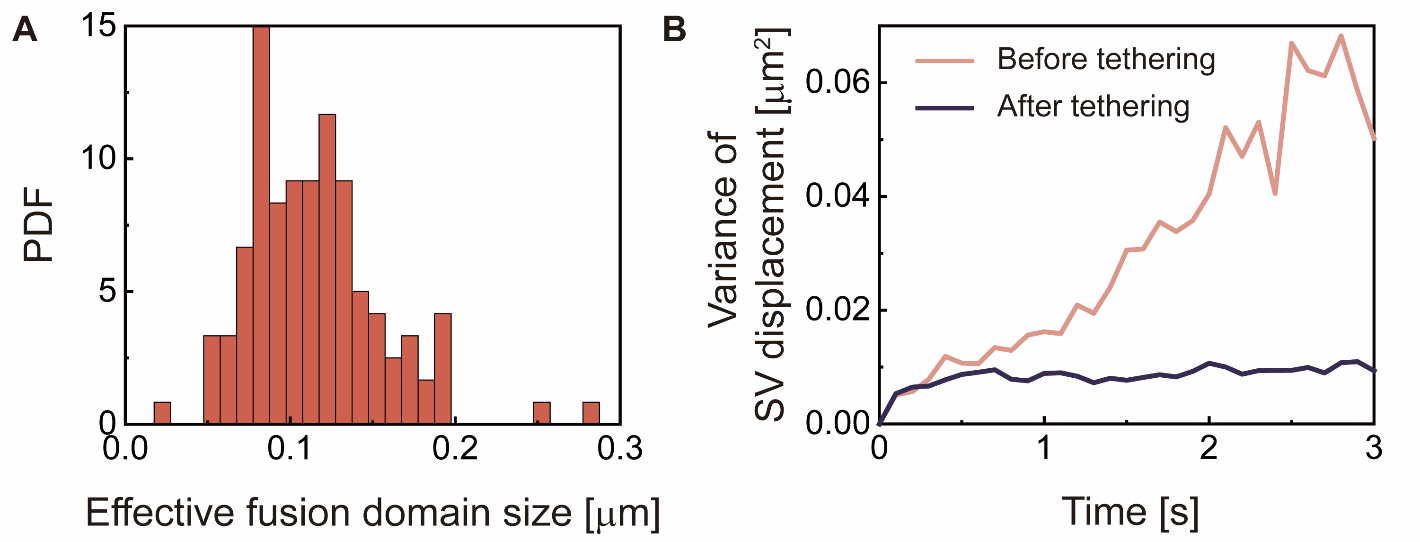


Figure S2. Effective fusion domain size and distinct motional characteristics of untethered and tethered synaptic vesicles. A) Distribution of the effective fusion domain size for a total of 120 SVs analyzed in this work. The effective fusion domain size is estimated as the radius of gyration, calculated from the outermost trajectory points constituting the convex hull, which encloses all points between the tethering and fusion events. B) Ensemble averaged variance of the SV displacement before and after the tethering event.


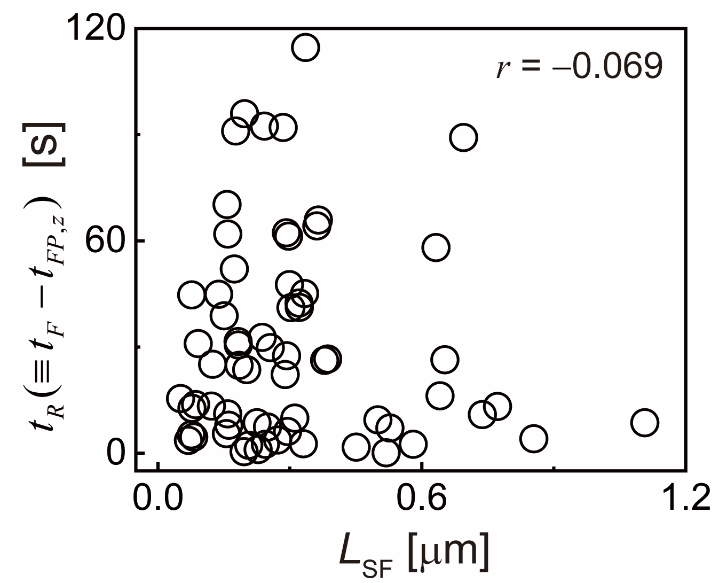


Figure S3. Weak correlation between the remaining time after the first passage event and the initial distance of Type II SVs. This figure shows that the remaining time, $t_{R}(\equiv t_{F}-t_{FP,z})$, defined as the difference between the fusion time $t_{F}$ and the first passage time $t_{FP,z}$, has only a weak correlation with the initial distance, $L_{SF}$, from the stimulation site to the fusion site for Type II SVs (*r* = −0.069).


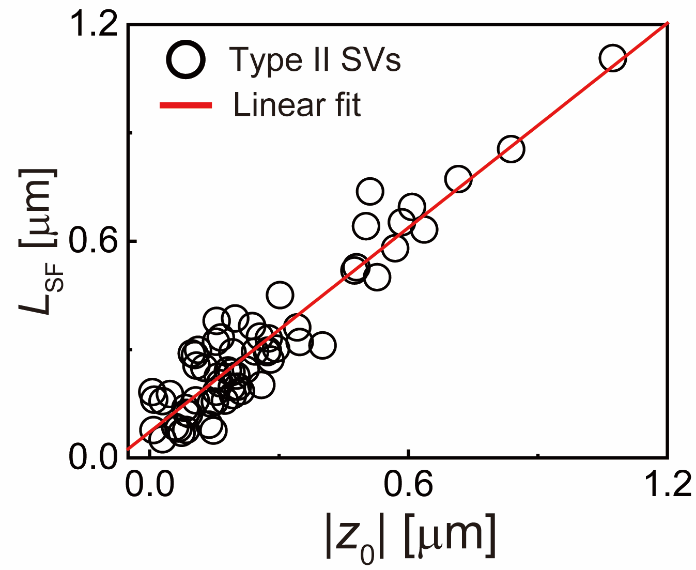


Figure S4. Linear relationship between two different initial distances of Type II SVs. This figure shows the linear relationship between the initial distance, $|z_{0}|$, from the stimulation site to the tethering site in the longitudinal direction and the initial distance, $L_{\text{SF}}$, from the stimulation site to the fusion site for Type II SVs (*r* = 0.94). The red solid line represents the best linear fit, given by $L_{\text{SF}}=A|z_{0}|+B$ with *A* = 0.943 and *B* = 7.18×10^−2^ μm.


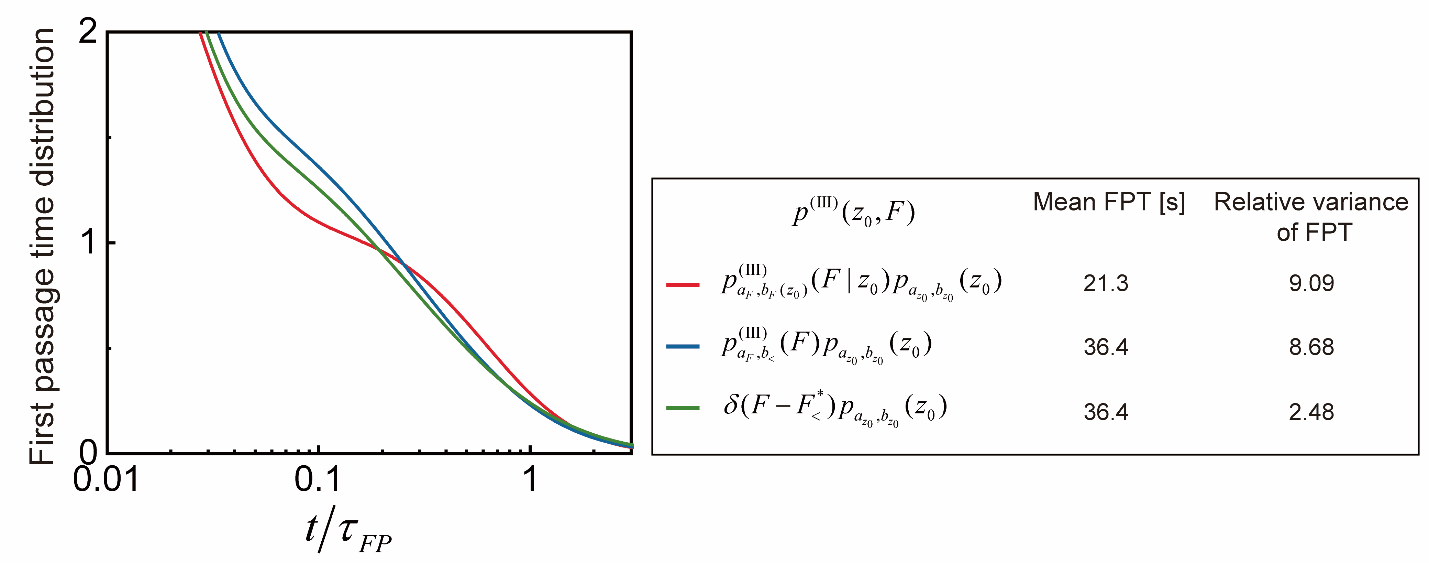


Figure S5. Effect of mean force heterogeneity on the first passage time distribution. The red line shows the first passage time (FPT) distribution obtained from the optimized Model III. The blue line represents the FPT distribution resulting from replacing the conditional probability distribution, $p_{a_{F},b_{F}(z_{0})}^{(\text{III})}(F|z_{0})$, in the optimized Model III by the mean force distribution, $p_{a_{F},b_{<}}^{(\text{III})}(F)$, with the initial distance ($|z_{0}|$)-independent scale parameter, $b_{F}(z_{0})=b_{<}$. The green line shows the FPT distribution obtained by further simplifying $p_{a_{F},b_{<}}^{(\text{III})}(F)$ to a delta-function distribution, $\delta(F-F_{<}^{*})$, centered at the mode, $F_{<}^{*}$, of $p_{a_{F},b_{<}}^{(\text{III})}(F)$. $\tau_{FP}$ denotes the mean first passage time in each case. Together with the relative variance of the FPT in each case, this figure suggests that the heterogeneity in the mean force contributes to the broadening of the fusion time distribution, as demonstrated by the FPT distribution. Note that the sigmoidal initial-distance dependence of the mean force reduces the mean first passage time to 21.3 s, about half of the value, 36.4 s, calculated for the case with an initial-distance independent distribution of the mean force, without introducing a significant change in the randomness of the first passage time.


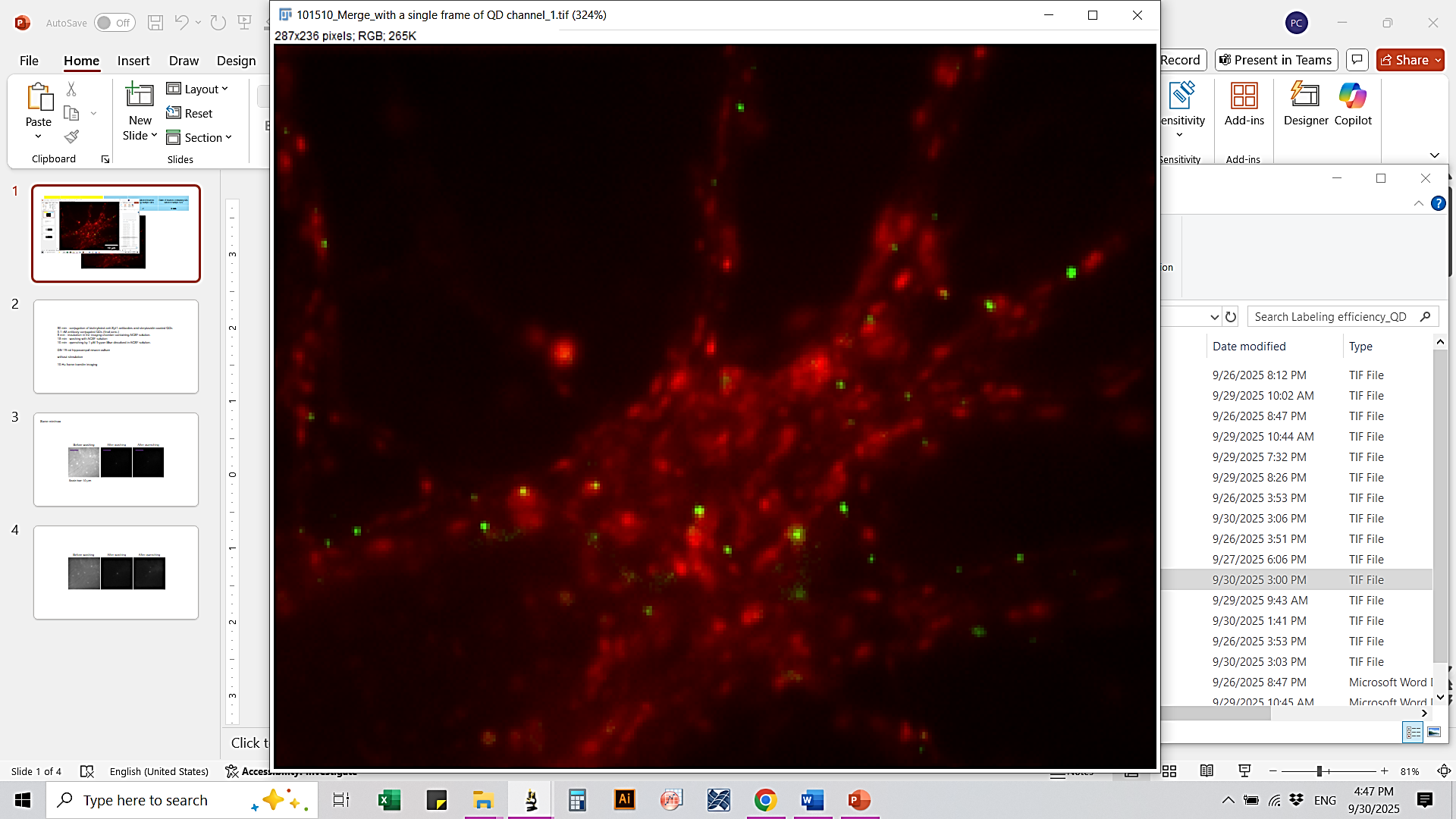


**Figure S6**. **Colocalized image of QDs (green) and boutons labeled with FM 4-64 (red) in the incubation of 0.1 nm of antibody-conjugated QDs.** QDs were sparsely labeled. Colocalized images showed around 20% (75 out of 375 bouton) of boutons had QDs. Scale bar represents 10 μm.

**Figure S7**. **One-step quenching of a single QD by trypan blue.** The graph of average fluorescence intensity in a region of interest (ROI) of a QD shows rapid irreversible one-step decrease in fluorescence intensity by quenching with trypan blue. This irreversible quenching indicates exocytosis of an SV containing a QD. Photoblinking indicates that this SV contained a QD.


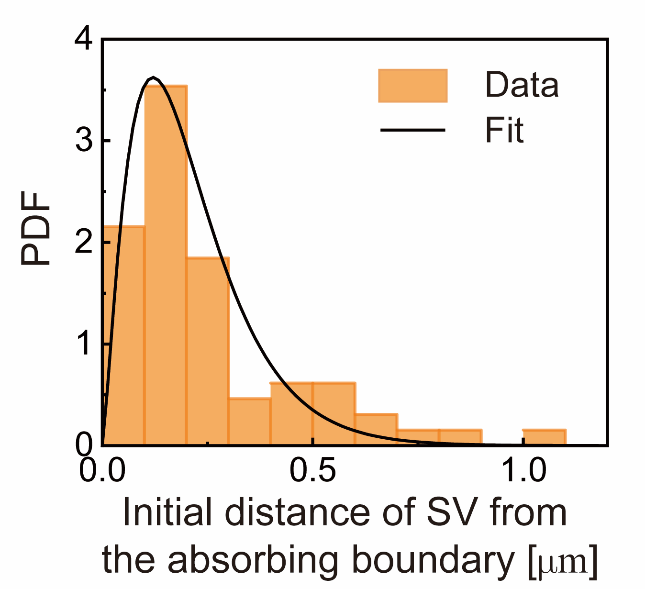


Figure S8. Distribution of initial longitudinal distances of synaptic vesicles from the absorbing boundary. The black line represents the best fit of a gamma distribution, $p_{a_{z_{0}},b_{z_{0}}}(z_{0})=|z_{0}|^{a_{z_{0}}-1}e^{-|z_{0}|/b_{z_{0}}}/\Gamma(a_{z_{0}})b_{z_{0}}^{a_{z_{0}}}$, to the experimental data (histogram) for the distribution of the initial synaptic vesicle distance, $z_{0}$, along the longitudinal *z*-axis from the absorbing boundary. The optimized values of $a_{z_{0}}$ and $b_{z_{0}}$ are given by $a_{z_{0}}=2.35$ and $b_{z_{0}}=8.92\times10$ nm.

| *α*-axis | $\varepsilon_{\alpha}$ (nm) | |
| --- | --- | --- |
|  | Pre-stimulation | Post-stimulation |
| Longitudinal *z*-axis | 16.6 | 29.2 |
| Transverse *x*-axis | 12.7 | 21.8 |
| Transverse *y*-axis | 17.5 | 30.3 |

**Supplementary Table S1. The mean jump length during the experimental time resolution** $\Delta t$**.** The mean jump length, $\varepsilon_{\alpha}$, is calculated as $\langle l_{\alpha}/t\rangle\Delta t$, where $l_{\alpha}$ represents the pathlength of a single SV trajectory along the *α*-axis during the time interval *t*. *t* corresponds to the time interval between the beginning of observation and the onset of stimulation in the pre-stimulation case, and the time interval between the onset of stimulation and the first passage event in the post-stimulation case.

Reference

1. S. O. Rizzoli, W. J. Betz, "Synaptic vesicle pools," *Nature Reviews Neuroscience,* (2005): *6* (1), 57.

2. D. Zenisek, J. Steyer, W. Almers, "Transport, capture and exocytosis of single synaptic vesicles at active zones," *Nature,* (2000): *406* (6798), 849.

3. J. Fieberg, C. O. Kochanny, "Quantifying home‐range overlap: the importance of the utilization distribution," *The Journal of Wildlife Management,* (2005): *69* (4), 1346.

4. A. Dani, B. Huang, J. Bergan, C. Dulac, X. Zhuang, "Superresolution imaging of chemical synapses in the brain," *Neuron,* (2010): *68* (5), 843.

5. T. Schikorski, C. F. Stevens, "Quantitative ultrastructural analysis of hippocampal excitatory synapses," *Journal of Neuroscience,* (1997): *17* (15), 5858.

6. R. Metzler, E. Barkai, J. Klafter, "Anomalous Diffusion and Relaxation Close to Thermal Equilibrium: A Fractional Fokker-Planck Equation Approach," *Physical Review Letters,* (1999): *82* (18), 3563.

7. H. Scher, E. W. Montroll, "Anomalous transit-time dispersion in amorphous solids," *Physical Review B,* (1975): *12* (6), 2455.

8. E. Barkai, "CTRW pathways to the fractional diffusion equation," *Chemical Physics,* (2002): *284* (1), 13.

9. E. W. Montroll, G. H. Weiss, "Random walks on lattices. II," *Journal of Mathematical Physics,* (1965): *6* (2), 167.

10. R. Metzler, E. Barkai, J. Klafter, "Deriving fractional Fokker-Planck equations from a generalised master equation," *Europhysics Letters,* (1999): *46* (4), 431.

11. A. V. Chechkin, F. Seno, R. Metzler, I. M. Sokolov, "Brownian yet Non-Gaussian Diffusion: From Superstatistics to Subordination of Diffusing Diffusivities," *Physical Review X,* (2017): *7* (2), 021002.

12. I. M. Sokolov, J. Klafter, "From diffusion to anomalous diffusion: a century after Einstein’s Brownian motion," *Chaos: An Interdisciplinary Journal of Nonlinear Science,* (2005): *15* (2), 026103.

13. S. Chandrasekhar, "Stochastic Problems in Physics and Astronomy," *Reviews of Modern Physics,* (1943): *15* (1), 1.

14. S. Redner, *A guide to first-passage processes*, (Cambridge University Press, 2001).

15. S. Song, S. J. Park, M. Kim, et al., "Transport dynamics of complex fluids," *Proceedings of the National Academy of Sciences,* (2019): *116* (26), 12733.

16. N. G. Van Kampen, *Stochastic processes in physics and chemistry*, (Elsevier, 1992).

17. R. Jordan, E. A. Lemke, J. Klingauf, "Visualization of synaptic vesicle movement in intact synaptic boutons using fluorescence fluctuation spectroscopy," *Biophysical journal,* (2005): *89* (3), 2091.

18. M. Shtrahman, C. Yeung, D. W. Nauen, G.-q. Bi, X.-l. Wu, "Probing vesicle dynamics in single hippocampal synapses," *Biophysical journal,* (2005): *89* (5), 3615.

19. C. Yeung, M. Shtrahman, X.-l. Wu, "Stick-and-diffuse and caged diffusion: a comparison of two models of synaptic vesicle dynamics," *Biophysical journal,* (2007): *92* (7), 2271.

20. G. E. Uhlenbeck, L. S. Ornstein, "On the theory of the Brownian motion," *Physical Review,* (1930): *36* (5), 823.
